# The impact of detention on the health of asylum seekers: An updated systematic review: A systematic review

**DOI:** 10.1002/cl2.1420

**Published:** 2024-07-08

**Authors:** Trine Filges, Elizabeth Bengtsen, Edith Montgomery, Malene Wallach Kildemoes

**Affiliations:** ^1^ VIVE Campbell VIVE – The Danish Centre of Applied Social Science Copenhagen Denmark; ^2^ Administration The Danish National Centre for Social Research Copenhagen Denmark; ^3^ Department of Public Health, Danish Research Centre for Migration, Ethnicity and Health University of Copenhagen Copenhagen Denmark

**Keywords:** field of practice, mental health, outcome, quantitative, study, systematic review

## Abstract

**Background:**

The number of people fleeing persecution and regional conflicts is rising. Western countries have applied increasingly stringent measures to discourage those seeking asylum from entering their country, amongst them, to confine asylum seekers in detention facilities. Clinicians have expressed concerns over the mental health impact of detention on asylum seekers, a population already burdened with trauma, advocating against such practices.

**Objectives:**

The main objective of this review is to assess evidence about the effects of detention on the mental and physical health and social functioning of asylum seekers.

**Search methods:**

Relevant literature was identified through electronic searches of bibliographic databases, internet search engines, hand searching of core journals and citation tracking of included studies and relevant reviews. Searches were performed up to November 2023.

**Selection criteria:**

Studies comparing detained asylum‐seekers with non‐detained asylum seekers were included. Qualitative approaches were excluded.

**Data collection and analysis:**

Of 22,226 potential studies, 14 met the inclusion criteria. These studies, from 4 countries, involving a total of 13 asylum‐seeker populations. Six studies were used in the data synthesis, all of which reported only mental health outcomes. Eight studies had a critical risk of bias. Meta‐analyses, inverse variance weighted using random effects statistical models, were conducted on post‐traumatic stress disorder (PTSD), depression, and anxiety.

**Main results:**

A total of 27,797 asylum seekers were analysed. Four studies provided data while the detained asylum seekers were still detained, and two studies after release. All outcomes are reported such that a positive effect size favours better outcomes for the non‐detained asylum seekers. The weighted average SMD while detained is 0.45 [95% CI 0.19, 0.71] for PTSD and after release 0.91 [95% CI 0.24, 1.57]; for anxiety 0.42 [95% CI 0.18, 0.66] and for depression 0.68 [95% CI 0.10, 1.26] both while detained. Based on single‐study data, the SMD was 0.60 [95% CI 0.02, 1.17] for depression and 0.76 [95% CI 0.17, 1.34] for anxiety, both after release. Three studies (one study each) reported outcomes related to psychological distress, self‐harm and social well being. Psychological distress favoured the detained but was not significant; whereas both effect sizes on self‐harm and social wellbeing indicated highly negative impacts of detention; in particular, the impact on self‐harm was extremely high. The OR of self‐harm was reported separately for asylum seekers detained in three types of detention: Manus Island, Nauru and onshore detention. The ORs were in the range 12.18 to 74.44; all were significant.

**Authors' conclusions:**

Despite similar post‐migration adversities amongst comparison groups, findings suggest an independent adverse impact of detention on asylum seekers' mental health, with the magnitude of the effect sizes lying in an important clinical range. These effects persisted beyond release into the community. While based on limited evidence, this review supports concerns regarding the detrimental impact of detention on the mental health of already traumatised asylum seekers. Further research is warranted to comprehensively explore these effects. Detention of asylum seekers, already grappling with significant trauma, appears to exacerbate mental health challenges. Policymakers and practitioners should consider these findings in shaping immigration and asylum policies, with a focus on minimising harm to vulnerable populations.

## 1 PLAIN LANGUAGE SUMMARY

### 1.1 Confining asylum seekers in detention centres negatively affects their mental health both during their detention and after their release

In this review, we aimed to find evidence of the impact of confining asylum seekers on their mental and physical health and social functioning.

### 1.2 What is this review about?

The number of people fleeing conflicts and persecution is increasing. However, many countries use harsh measures to discourage people who wish to apply for asylum, including confining asylum seekers in detention centres. The number of such centres is rising. Understanding the health impact of detaining asylum seekers is important. Asylum seekers have high rates of pre‐migration trauma from exposure to war, genocide or imprisonment. These experiences make them vulnerable to health problems. Confining them may worsen the effects of the trauma they have experienced already.

This Campbell systematic review assessed whether detaining asylum seekers has an impact on their mental health. The review also assessed whether detaining asylum seekers has a negative impact on their social functioning.

### 1.3 What is the aim of this review?

We aimed to examine the impact of detaining asylum seekers on their mental health, physical health and social functioning.

### 1.4 What studies are included?

Included studies compared asylum seekers who were detained with those who were not detained.

A total of 14 studies met the requirements for inclusion. The studies were conducted in four countries: the UK, Japan, Canada, and Australia.

All the studies used non‐randomised designs. Eight of the studies were excluded from the analysis because there were important differences between the groups which were compared, or because the studies were judged to have methodological limitations. All the excluded studies were conducted in Australia, which has a policy of mandatory detention.

### 1.5 What is the impact?

Detention has a negative impact on the mental health of asylum seekers. Levels of posttraumatic stress disorder (PTSD), depression, and anxiety both before and after release were found to be higher amongst asylum seekers who were detained compared to those who were not detained. The size of the effects was clinically important. One study each reported outcomes related to psychological distress, social functioning and self‐harm. In particular self‐harm was highly related to detention.

### 1.6 What do the findings of this review mean?

Policymakers should consider less harmful policy options than detention. These options may include reporting requirements, sureties or bail, or community supervision. Options that restrict people's freedom of movement should also be closely monitored to ensure that these do not also have negative mental health effects.

The research summarised in the review is of moderate quality. Further research is needed to assess the impacts of keeping asylum seekers in detention centres on their physical and mental health and social functioning. A deeper, comparative understanding of the impacts of different detention conditions on asylum seekers is also needed.

## 2 BACKGROUND

### 2.1 The problem, condition or issue

The last decades of the twentieth century were accompanied by an upsurge in the number of persons fleeing persecution and regional wars. According to the statistics offered by the United Nations High Commissioner for Refugees (UNHCR) 1,262,649 asylum applications were received by the countries in Europe, Canada, USA, Japan, Australia and New Zealand in 2022 (see https://www.unhcr.org/refugee-statistics/download/?url=1p7ePZ). Eurostat provides statistics on the gender and age distribution of asylum seekers in the EU. The most recent data is from 2022 where males account for 71%; children under 18 years, 25%; those aged 18–34 years, 54%; and those 35 years and older, 21% (see https://doi.org/10.2908/MIGR_ASYAPPCTZA).

Western countries have applied increasingly stringent measures to discourage those seeking asylum from entering their countries (United Nations, 2000; Human Rights Watch, 2001). There are various strategies aimed at deterring the influx of asylum seekers. These include confinement in detention centres, enforced dispersal within the community, more stringent refugee determination procedures, and temporary forms of asylum. In several countries, asylum seekers living in the community face restricted access to work, education, housing, welfare, and in some situations, to basic health care services (Silove, 2000).

The most controversial of the measures to discourage people from seeking asylum is the decision by some Western countries to confine asylum seekers in detention facilities (Loff, 2002; Summerfield et al., 1991). Many countries detain asylum seekers; however, Australia has been unique in establishing a policy of mandatory, indefinite detention. From 1992 to 2005, Australia implemented a policy of mandatory detention of all asylum seekers arriving by boat or without valid travel documents. This policy has been much criticised (Janet & Harriet, 2013) and in November 2011, Australia changed its policy aimed at limiting the time asylum seekers are held in detention (Cleveland et al., 2012b). In 2013 the Australian government announced a policy in which any asylum seeker arriving by boat without a visa will be refused settlement in Australia, instead they will be settled in Papua New Guinea (PNG) if they are found to be legitimate refugees (Regional resettlement arrangement between Australia and Papua New Guinea, 2013, National Legislative Bodies/National Authorities, 2013). The UNHCR has expressed concern with the policy, especially the lack of national capacity and expertise in processing, and poor physical conditions within open‐ended, mandatory and arbitrary detention settings (United Nations High Commissioner for Refugees, 2013).

Since the events of 9/11, other countries such as the USA and the UK (American Civil Liberties Union ACLU, 2007; Michael & Liza, 2005) have expanded immigration detention facilities and the use of detention. A similar trend appears to have emerged in Canada (Lacroix, 2006; NYERS, 2003). In December 2012 Canada implemented changes to the refugee determination system inter alia implying that asylum seekers aged 16 or older and designated as part of an ‘irregular arrival’ will be detained (Cleveland et al., 2012b; Canadian Council for Refugees, 2012). Furthermore, in a number of continental European countries, the use of detention has significantly increased and is often used as a first resort rather than last resort (Council of Europe, 2010).

Asylum seekers are detained at different stages of the asylum process. Detention is also used by most European countries to facilitate deportations (Schuster, 2004). Hence, recently arrived asylum seekers as well as asylum seekers whose appeals have not yet been heard are held in detention. In many European countries, deportation orders are issued concurrently with the initial rejection of the asylum claim (Schuster, 2004; Hughes & Liebaut, 1998).

Since the events of 9/11, other countries such as the USA and the UK (American Civil Liberties Union ACLU, 2007; Michael & Liza, 2005) have expanded immigration detention facilities and the use of detention. A similar trend appears to have emerged in Canada (Lacroix, 2006; NYERS, 2003). In December 2012, Canada implemented changes to the refugee determination system inter alia, implying that asylum seekers aged 16 or older and designated as part of an ‘irregular arrival’ will be detained (Cleveland et al., 2012b; Canadian Council for Refugees, 2012). Furthermore, in a number of continental European countries, the use of detention has significantly increased and is often used as a first resort rather than last resort (Council of Europe, 2010).

Asylum seekers are detained at different stages of the asylum process. Detention is also used by most European countries to facilitate deportations (Schuster, 2004). Hence, recently arrived asylum seekers as well as asylum seekers whose appeals have not yet been heard are held in detention. In many European countries, deportation orders are issued concurrently with the initial rejection of the asylum claim (Schuster, 2004); Hughes & Liebaut, 1998).

There are no official statistics on how many asylum seekers are detained or for how long (Hughes & Liebaut, 1998; Human Rights and Equal Opportunity Commission, 1998; The Information Centre about Asylum and Refugees [ICAR], 2007). A few countries do provide some information regarding the number and duration of detention of asylum seekers, however. In Australia, immigration detention statistics are provided by the Department of Home Affairs. Here, the statistic is given as a monthly snapshot on a particular date as opposed to a general annual total. As of 31 May 2013, there were 8521 persons in immigration detention facilities (including alternative places of detention) of which 79% were males and 18% were children (less than 18 years of age). There has been a significant decrease in the number of people in immigration detention facilities since then. In February 2024 there were 881 (including 785 with a criminal history) people in immigration detention facilities of which 93.5% were male, 5.8% female and 0.7% children (less than 18 years of age). The average duration of detention in Australia is likewise given only as a snapshot, and calculated as the average length of time (so far) for persons held in detention on a particular date. Thus, no statistics are published of the overall periods spent in detention by each detainee. Contrary to the number of people detained, the snapshot average length has increased from 74 days as of 31 May 2013 to 624 days as of February 2024. The length of stay in 2024 varied from 7 days or less (5.3%) to more than 1825 days (8.6%). The majority (19.2%) had spent between 183 and 365 days in detention. In the UK, the Home Office provides statistics, as of 31 December 2012, there were 1676 asylum seekers in detention, decreasing slightly to 1317 as of June 2022. The length of stay is not provided separately for immigrants who have sought asylum.

Little is known about why people are detained. There is no accessible legal framework governing the use of detention under either international human rights law or refugee law. According to the Council of Europe (2010), the national laws and regulations of many countries are insufficient and leave too much at the discretion of immigration officials. Detention policies are non‐transparent, which may imply a certain degree of arbitrariness in the decision process (Council of Europe, 2010).

Since 1999, UNHCR Guidelines (UN High Commissioner for Refugees UNHCR, 1999) have suggested considering the following as possible alternatives to detention monitoring requirements: provision of a guarantor/surety, release on bail, and open centres (JRS Europe policy). There are many ways in which these alternatives to detention are implemented in practice. JRS Europe (Jesuit Refugee Service Europe) emphasises that the type of alternative to detention that a government uses must fit the country's particular context, and especially the needs of the migrants who are participating in that alternative (Jesuit Refugee Service Europe, 2013).

That the decision to detain is often arbitrary is also stated by the UNHCR: ‘In many States the decision to detain is taken on the basis of sometimes very wide discretionary powers, often not prescribed by law. Moreover, even when the grounds upon which such orders are made are established in law, these are far too frequently applied in an arbitrary manner’ (United Nations High Commissioner for Refugees, 1999a, p. 3).

Although UNHCR guidelines on the detention of asylum seekers include the right to an automatic independent judicial review of all decisions to detain followed by periodic reviews of the necessity to continue to detain, several member states do not comply with UNHCR's guidelines on the detention of asylum seekers (United Nations, 2000; Human Rights Watch, 2001).

There is, however, growing evidence that the detention of asylum seekers is associated with substantial mental health problems (Mina & Derrick, 2006; Derrick et al., 2001; Physicians for Human Rights and the Bellevue/NYU Programme for Survivors of Torture, 2003). The Bellevue/NYU Programme for Survivors of Torture (Bellevue/NYU) and Physicians for Human Rights study reports that significant symptoms of depression were present in 86% of the detained asylum seekers; anxiety was present in 77% and PTSD in 50%. Hence, the mental health of asylum seekers was extremely poor and worsened the longer these individuals were in detention.

One important question arises from this: Is there any evidence of a causal effect of detention on the mental problems of asylum seekers? Research using appropriate controls can provide some relevant evidence on whether detention might cause adverse outcomes for asylum seekers: Considering the particular population under investigation in this review, it is vital that an appropriate comparison group is used to establish causality.

Another concern is that diagnostic difficulties can arise in a multicultural context, particularly when applying some Western mental health diagnoses to other cultures.

The ways of expressing distress and views on the causes of that distress may differ markedly from those of the dominant ‘Western’ culture. For example, depression may be seen as the result of ‘thinking too much’ or of witchcraft (Patel, 1995; Vikram et al., 1995). Some ethnic groups do not have certain Western diagnostic concepts, such as alcoholism, in their vocabulary, and the stigma attached to mental illness in some cultures may even be greater than in Western society (Jo & Rachel, 2002). Furthermore, although similar symptoms may exist in different cultures, they do not necessarily have the same value or meaning and there is variation in what is understood to constitute ‘normal’ emotional expression. For example, in some cultures, dreams of the dead are perceived as positive and comforting (Zur, 1996). Kirmayer (1996) discusses differences between cultures in how conscious and non‐conscious ways of dealing with distress are promoted, and notes that intrusion and avoidance symptoms vary in their ‘normality’ across cultures.

Asylum seekers often come from countries in conflict and many asylum seekers have experienced pre‐migration adversities that may have affected their health (Silove, 2000; Katy et al., 2009). High rates of pre‐migration trauma, and therefore of trauma‐related mental health problems, have been reported (Ingrid et al., 1997). However, research into post‐migration adversities suggests that aspects of the asylum‐seeking process may compound the stressors suffered by an already traumatised group (Sinnerbrink et al., 1997). Similarly, Derrick et al. (1997) conclude: ‘Our findings raise the possibility that current procedures for dealing with asylum‐seekers may contribute to high levels of stress and psychiatric symptoms in those who have been previously traumatised’, (Derrick et al., 1997, p. 351). Seven common post‐migration adversities are identified (termed the ‘seven Ds’): Discrimination, Detention, Dispersal, Destitution, Denial of the right to work, Denial of healthcare, and Delayed decisions on asylum applications (see Helen et al., 2008).

Hence, as detention is not the only post‐migration stressor and considering the fact that the population under investigation in this review most likely has high rates of pre‐migration trauma; we believe it is vital that an appropriate comparison group is used to establish causality. In particular, the comparison group should have similar rates of pre‐migration trauma (and time to recover in the country where asylum is sought) and be of the same geographical/ethnic orientation.

The main objective of this review is to assess what is known about the causal effects of detention on asylum seekers' mental health. The aim is to uncover and synthesise relevant studies that measure the causal effects on mental health of detaining asylum seekers. Although the primary focus is on mental health, all outcomes reported in studies comparing detained asylum seekers with a comparable non‐detained group are examined.

We are aware that tight causal conclusions cannot be drawn from the studies we found, as none were based on trials. However, a distinction can be drawn between studies that simply assess the association between the detention of asylum seekers and mental health outcomes, and studies that control for important confounding factors. Studies that control for important confounding factors provide some evidence for considering possible causal effects (See Section Selection criteria, 4 for a discussion of confounding factors). While conclusions about causal effects must be very tentative, it is important to extract and summarise the best evidence available.

### 2.2 The intervention

In this review, the detention of asylum seekers is regarded as a social intervention – with possible adverse consequences for the asylum seekers. A report from the Human Rights and Equal Opportunity Commission (1998) argues that detention of asylum seekers breach international human rights standards; seeking asylum is not illegal under international law and people have a right to be treated humanely and with dignity.

We define detention as the deprivation of liberty for asylum seekers in the host country**.** Those detained may be held in various facilities (immigration holding centres, remote camps or provincial jails) which may be run by public authorities or by private companies. In most countries, the detention of asylum seekers is an administrative procedure that is undertaken to verify the identity of individuals, process asylum claims, and/or ensure that a deportation order is carried out (The Global Detention Project, www.globaldetentionproject.org). It is important to note that one of the key concerns vis‐à‐vis this form of detention is precisely its administrative nature. Domestic legal systems are rarely detailed regarding these detention situations, which can result in detainees facing legal uncertainty (including lack of access to the outside world, e.g., to legal counsel), inadequate or no possibilities of challenging detention through the courts, and lack of limitations on the duration of detention. Living conditions differ, but in many countries, detention centres are operated as if they were prisons, with barred windows, high‐wire perimeter fencing, and with limited access to information, health care services and psychological support (The Global Detention Project and [Amaral & Jesuit Refugee Service Europe, 2010]).

### 2.3 How the intervention might work

Asylum seekers who are detained in the host country experience a set of stressors, reflecting the detention process itself and the detention centre environment, which may adversely affect their mental health status. These include loss of liberty, uncertainty regarding return to their country of origin, uncertain duration of detention, social isolation, separation from families, abuse from staff, riots, forceful removal, hunger strikes, and self‐harm (Mina & Derrick, 2006; Keller et al., 2003; Pourgourides et al., 1996).

How the mental health status of detained asylum seekers after release relates to the nature of their experience of detention has rarely been subjected to detailed examination and only a few such studies exist.

In the Bellevue/NYU Programme for Survivors of Torture (Bellevue/NYU) and Physicians for Human Rights study (Physicians for Human Rights and the Bellevue/NYU Programme for Survivors of Torture, 2003), it is reported that confinement and the loss of liberty profoundly disturbed asylum seekers and triggered feelings of isolation, powerlessness and disturbing memories of persecution that asylum seekers had suffered in their countries of origin. The study by Amaral (Amaral & Europe, 2010) shows that detention and the negative factors associated with it has a significant deteriorative effect on asylum seekers' self‐perception, with minors and long‐term detainees appearing to suffer the most.

Further research was undertaken in the Coffey et al. (2010) study, to examine the experience of detention from the perspective of the detained asylum seekers, and to identify the consequences of these experiences for their life after release. Detention was experienced as a dehumanising environment characterised by confinement, deprivation, injustice, inhumanity, isolation, fractured relationships, and mounting hopelessness and demoralisation.

The probable mechanisms by which the harmful effects of detention were transmitted appear to include the following: Changes in self‐perception, changes in relationships in accordance with how the detainee was perceived and treated by others and by ‘the system’, and alteration of core values. These mechanisms are recognised in psychological literature, especially in the trauma field, as ways in which negative psychological effects are maintained following experiences which threaten the self (Lewis, 1997; Abernathy, 2008; Janoff‐Bulman, 1992; Lifton, 1993; Keith et al., 2004).

Certain types of people are regarded as being vulnerable, that is, they may be especially susceptible to harm in detention. Women, children, unaccompanied minors and persons with a mental or physical disability are widely acknowledged to be vulnerable (Amaral & Europe, 2010). Amaral defines vulnerability as a ‘loss of control over oneself to someone, or something, with more power, thus making oneself susceptible to some type of harm’ (Amaral & Europe, 2010, p. 94). He concludes that the lack of information regarding asylum procedures, duration and reasons for detention and expected release is a critical indicator of detainees' ability to cope with their time in detention. According to Amaral (2010), younger detainees aged 10 to 24 are reported to possess less information compared to older detainees. Women in general, but especially women aged 18–24, are reported to possess less information than men do. Thus, younger detainees, and especially younger women, seem to particularly suffer from detention.

The UNHCR definition of vulnerable groups, in addition to the ones mentioned above, includes torture or trauma victims (United Nations High Commissioner for Refugees, 1999b).

This points towards another important aspect of the probable mechanisms by which detention may adversely affect detainees. Research suggests that asylum seekers worldwide report high rates of pre‐migration trauma and adversities (e.g., war, imprisonment, genocide, physical and sexual violence, witnessing violence to others, traumatic bereavement, starvation and homelessness) (Sinnerbrink et al., 1997; McColl et al., 2008), and therefore of trauma‐related mental health problems. The process of seeking asylum in Western countries places additional demands on this group. Post‐migratory stressors, in particular detention, seem to negatively affect this population, who are already vulnerable to mental health difficulties as a result of their previous exposure to traumatic events. Even though captivity is stressful in any context and in particular when it occurs over an indeterminate period, it may be even more stressful for people who have had previously traumatic experiences (Jo & Rachel, 2002; Pourgourides, 1997). The experience of detention may reactivate and exacerbate previous trauma. For example, the Medical Foundation for the Care of Victims of Torture (1994) reports that the indeterminate detention experienced by asylum seekers who have previously been imprisoned and tortured may prolong the psychological ‘demolition’ of the person and cause high levels of stress, despair and anxiety.

### 2.4 Why it is important to do this review

Given the well‐documented vulnerability of asylum seekers as a result of traumatic experiences before arrival, a number of clinicians have expressed concern that detention increases mental health difficulties in adult and child asylum seekers, and have called for an end to such practices (Fazel & Stein, 2004; Salinsky, 1997; Koopowitz & Abhary, 2004). This is clearly in conflict with government policies aimed at reducing the numbers of asylum seekers (Silove, 2000).

An obvious question arises: Is it worth conducting a systematic review when the likelihood is that few trial‐based studies are expected to be found? We believe so, as a systematic review may uncover high quality studies that may not be found using less thorough search methods. Secondly, if a systematic review demonstrates that high quality studies are lacking, this could encourage a new generation of primary research. Hence, even though we did not expect to find any trial‐based studies (and did not find any) and very few studies of the detention of asylum seekers based on control group comparison, we still believe it is worth conducting a review to gather and highlight the best available knowledge.

## 3 OBJECTIVES

The main objective of this review is to assess evidence about the effects of detention on the mental and physical health and social functioning of asylum seekers.

## 4 METHODS

The title for this systematic review was registered in December 2012. The systematic review protocol was approved on November 27, 2013 and published on 02.01.2014 (Filges et al., 2014). The original review was published with the Campbell Collaboration in 2015 and as an invited journal article in 2018 (published online in 2916) (Filges et al., 2015, 2018).

### 4.1 Criteria for considering studies for this review

#### 4.1.1 Types of studies

Due to ethical considerations, it is hard to imagine that a researcher would control the allocation of asylum seekers into detention and non‐detention conditions. We therefore anticipated that relatively few controlled trials on this topic would be found although, in the unlikely event that a controlled trial had been found, it would have been included in the review. To summarise what is known about the possible causal effects of detention, we included all study designs that used a well‐defined control group such as, for example, asylum seekers in the same country who are not detained. Non‐randomised studies, where the use of detention occurred in the course of usual decisions outside the researcher's control, must have demonstrated pretreatment group equivalence via matching, statistical controls, or evidence of equivalence in the magnitude of key risk variables and participant characteristics. These factors are outlined in Section 4.3.3.1, and the methodological appropriateness of the included studies was assessed according to the risk of bias model outlined in Section 4.3.3.1.

The study designs eligible for inclusion in the review were:

1. Controlled trials (where all parts of the study are prospective, such as identification of participants, assessment of baseline, and allocation to intervention which may be randomised, quasi‐randomised or non‐randomised), assessment of outcomes and generation of hypotheses (Higgins & Sally, 2011).
2. Non‐randomised studies where the use of detention has occurred in the course of usual decisions, the allocation to detention and non‐detention is not controlled by the researcher, and there is a comparison of *two or more groups* of participants. In non‐randomised studies, participants are allocated by means such as time differences, location differences, decision makers or policy rules.

#### 4.1.2 Types of participants

The ‘intervention population’ comprised asylum seekers who had been detained. The comparison population comprised asylum seekers who had not been detained. Asylum seekers whose asylum application had not been successful were included. We included asylum seekers of all ages and nationalities.

According to the United Nations Convention relating to the Status of Refugees as amended by its 1967 Protocol (the Refugee Convention, 1967), a refugee is a person who is outside their own country and is unable or unwilling to return due to a well‐founded fear of being persecuted because of their race, religion, nationality, membership of a particular social group, or political opinion (United Nations High Commissioner for Refugees, 2010). The terms ‘asylum seeker’ and ‘refugee’ are often used interchangeably. We follow UNHCR's definition and use the term ‘asylum seeker’ to mean an individual who has sought international protection and whose claim for refugee status has not yet been determined. As part of its obligation to protect refugees on its territory, the country of asylum is normally responsible for determining whether an asylum‐seeker is a refugee or not. This responsibility is often incorporated in the national legislation of the country and, for State Parties, is derived from the 1951 Convention Relating to the Status of Refugees (United Nations High Commissioner for Refugees, 2010). Only after the recognition of the asylum seeker's protection needs, can he or she officially be referred to as a refugee and enjoy refugee status, which carries certain rights and obligations according to the legislation of the receiving country.

#### 4.1.3 Types of interventions

The intervention is the detention of asylum seekers, defined as the deprivation of liberty (personal freedom being taken away) for asylum seekers in the host country. Studies investigating returned asylum seekers detained in their home country (due to having applied for asylum) were not included. In most countries, the detention of asylum seekers is an administrative procedure and domestic legal systems rarely detail the detention situations. Detention of asylum seekers may be undertaken to verify the identity of individuals, process asylum claims, and/or ensure that a deportation order is carried out.

#### 4.1.4 Types of outcome measures

We planned to include and examine all outcomes (such as mental health, physical health and social functioning) reported in studies using a comparable control group, although our primary focus was on measures of mental health.

#### 4.1.5 Duration of follow‐up

Time points planned for measures were:

- For participants currently detained
- From the end of detention to 1 year after release
- More than 1 year after release

No studies provided data more than 1 year after release.

#### 4.1.6 Types of settings

All types of settings were eligible. The detained may be held in various detention facilities such as immigration holding centres, remote camps or provincial jails which may be run by public authorities or private companies.

### 4.2 Search methods for identification of studies

Search strategies for the original version of this review were reported in (Filges et al., 2015). The search for the update was performed by two review authors (EB, TF) of which one (EB) is an information specialist. We followed the search strategy of the original review.

Relevant studies for the update were identified through electronic searches in bibliographic databases, grey literature repositories and resources, citation tracking, contact to international experts and Internet search engines. Since we already searched the literature with no date restrictions from November 2013 through April 2014 in the original review, a date restriction of 2014 and onwards was applied in the updated search. No language restrictions were applied to the searches.

#### 4.2.1 Electronic searches

The following electronic bibliographic databases were searched:

- APA PsycINFO (EBSCO) – October 2023
- PTSDpubs (ProQuest) – November 2023
- International Bibliography of the Social Sciences (ProQuest) – November 2023
- MEDLINE (OVID) – November 2023.
- PubMed – November 2023
- SocINDEX (EBSCO) – October 2023
- Academic Search Premier (EBSCO) – October 2023

The database searches were performed between 25/10/2023 and 14/11/2023.

##### Description of the search‐string

The search string is based on the PICO(s)‐model, and contains two concepts, of which we developed two corresponding search facets: population characteristics and the intervention. The search string includes searches in title and abstract as well as subject terms and/or keywords for each facet. The subject terms in the facets were selected according to the thesaurus or index of each database. Keywords were supplied where the search technique provided additional results. Use of truncation and wildcards were used to address English spelling variants.

##### Example of a search‐string

Below is an exemplified search string utilised to search MEDLINE through the OVID search interface and exemplifies the search facets as they were searched:

#### 4.2.2 MEDLINE (OVID)

Search strategy November 2nd, 2023.

Limit: 2014–2023

**Table:** 

#	Query	Results
**1**	(asylum adj1 seek*).ab,ti.	2638
**2**	(Asylumseeker* or Asylum‐seeker*).ab,ti.	2291
**3**	‘Asylum applicant*’ .ab,ti.	39
**4**	(Asylum adj1 claim*).ab,ti.	61
**5**	‘Exile*’ .ab,ti.	704
**6**	‘Fugitive*’ .ab,ti.	886
**7**	‘Displaced person*’ .ab,ti.	908
**8**	(Refuge* or Migrant* or Immigrant*).ab,ti.	66,575
**9**	Refugees.sh.	13,405
**10**	1 or 2 or 3 or 4 or 5 or 6 or 7 or 8 or 9	72,052
**11**	detention.ab,ti.	3733
**12**	‘confin*’ .ab,ti.	120,905
**13**	(Depriv* adj2 liberty).ab,ti.	282
**14**	(Detain or Detained).ab,ti.	1992
**15**	(Restrain or Restrained).ab,ti.	21,739
**16**	(Confine or confined).ab,ti.	92,202
**17**	Immigration holding.ab,ti.	1
**18**	‘Imprison*’ .ab,ti.	2947
**19**	‘Incarcerat*’ .ab,ti.	14,948
**20**	(Reception adj1 cent*).ab,ti.	276
**21**	(Asylum adj1 cent*).ab,ti.	79
**22**	(Accommodation adj1 cent*).ab,ti.	54
**23**	Temporary protection.ab,ti.	216
**24**	Retention.ab,ti.	217,007
**25**	(refugee adj1 camp*).ab,ti.	1514
**26**	‘custod*’ .ab,ti.	4976
**27**	(Prison* or jail*).ab,ti.	22,659
**28**	11 or 12 or 13 or 14 or 15 or 16 or 17 or 18 or 19 or 20 or 21 or 22 or 23 or 24 or 25 or 26 or 27	402,134
**29**	10 and 28	3432
**30**	29 and 2014:2023.(sa_year).	1939

The documentation of the search strategies from the remaining databases can be found in Supporting Information: 1.

#### 4.2.3 Searching other resources

##### Hand‐Search

The following journals that we considered most likely to include relevant primary studies were hand searched for the years 2023 and 2024:

- *Journal of Refugee Studies*
- *International Migration Review*
- *Forced Migration Review*
- *International Migration*
- *Refugee*

###### Grey literature searches

We used Google and Google Scholar search engines and the advanced search options to search the web to identify potential studies which were unpublished and/or in progress. We checked the first 200 hits. Moreover, we searched WHO Europe, WHO Western Pacific, WHO Americas, the World Bank, Amnesty International, SSRN.

##### Citation‐tracking

To identify both published studies and grey literature, we utilised citation‐tracking/snowballing strategies. Our primary strategy was to citation‐track related systematic‐reviews and meta‐analyses. The review team also checked reference lists of included primary studies for new leads.

##### Contact to experts

By e‐mail during November 2023, we contacted international experts to identify unpublished and ongoing studies.

### 4.3 Data collection and analysis

#### 4.3.1 Selection of studies

In pairs of two, two review authors (TF, MWK) and one research assistant (FMGB) independently screened titles and abstracts to exclude studies that were clearly irrelevant. Studies considered eligible by at least one author or studies where there was insufficient information in the title and abstract to judge eligibility, were retrieved in full text. The full texts were then screened independently in pairs of two, by two review authors (TF, MWK) and one research assistant (FMGB). Any disagreement about eligibility was resolved by discussion. Exclusion reasons for studies that otherwise might be expected to be eligible are documented.

The study inclusion criteria were identical to the ones used in Filges et al. (2015). The overall search and screening process is illustrated in a flow diagram. None of the review authors were blind to the authors, institutions, or the journals responsible for the publication of the articles.

#### 4.3.2 Data extraction and management

In pairs of two, review authors independently coded and extracted data from all the included studies. Except for the risk of bias coding sheet, the coding sheets were identical to the ones used in (Filges et al., 2015). Disagreements were minor and resolved by discussion. Data and information was extracted on: available characteristics of participants, intervention characteristics and control conditions, research design, sample size, risk of bias and potential confounding factors, outcomes, and results. Analysis was conducted using RevMan Web. Extracted numerical and descriptive data, and the risk of bias assessments described in the next section, can be found in the Supporting Information.

#### 4.3.3 Assessment of risk of bias

We updated our approach to the assessment of risk of bias from the previous review (Filges et al., 2015), to incorporate more explicit methods that had been developed since the original review was conducted.

We assessed the risk of bias in non‐randomised studies, using the model ROBINS–I, developed by members of the Cochrane Bias Methods Group and the Cochrane Non‐Randomised Studies Methods Group (Sterne et al., 2016). We used the latest template for completion (currently it is the version of 19 September 2016).

The ROBINS‐I tool is based on the Cochrane RoB2 tool for randomised trials, which was launched in 2008 and modified in 2011 (Higgins et al., 2011).

The ROBINS‐I tool covers seven domains (each with a set of signalling questions to be answered for a specific outcome) through which bias might be introduced into non‐randomised studies:

(1) bias due to confounding;
(2) bias in selection of participants;
(3) bias in classification of interventions;
(4) bias due to deviations from intended interventions (separate signalling questions for effect of assignment and adhering to intervention);
(5) bias due to missing outcome data;
(6) bias in measurement of the outcome;
(7) bias in selection of the reported result.

The first two domains address issues before the start of the interventions and the third domain addresses classification of the interventions themselves. The last four domains address issues after the start of interventions and there is substantial overlap between these four domains between bias in randomised studies and bias in non‐randomised studies trials (although signalling questions are somewhat different in several places, see Sterne, Higgins, et al., 2016 and Higgins et al., 2019).

Non‐randomised study outcomes were rated on a ‘Low/Moderate/Serious/Critical/No Information’ scale in each domain. The level ‘Critical’ means: the study (outcome) is too problematic in this domain to provide any useful evidence on the effects of intervention, and it is excluded from the data synthesis.

We stopped the assessment of a non‐randomised study outcome as soon as one domain in the ROBINS‐I was judged as ‘Critical’.

‘Serious’ risk of bias in multiple domains in the ROBINS‐I assessment tool may lead to a decision of an overall judgement of ‘Critical’ risk of bias for that outcome, and it will be excluded from the data synthesis.

##### Confounding

An important part of the risk of bias assessment of non‐randomised studies is how the studies deal with confounding factors. Selection bias is understood as systematic baseline differences between groups and can therefore compromise comparability between groups. Baseline differences can be observable (e.g., age and gender) and unobservable (to the researcher; e.g., ‘appearance’ of the asylum seeker). There is no single non‐randomised study design that always deals adequately with the selection problem: different designs represent different approaches to dealing with selection problems under different assumptions and require different types of data. There can be considerable variation in how different designs deal with selection on unobservables. The ‘adequate’ method depends on the model generating participation, that is, assumptions about the nature of the process by which participants are selected into a programme.

The primary studies must have demonstrated pretreatment group equivalence via matching, statistical controls, or evidence of equivalence on key risk variables and participant characteristics.

For this review, we identified the following observable confounding factors as most relevant: prior trauma exposure, gender, age, time since arrival to the country where asylum is applied for, and geographical/ethnic orientation. In each study, we assessed whether these confounding factors had been considered. We also assessed other confounding factors considered in the individual studies, and assessed how each study dealt with unobservables.

##### Importance of pre‐specified confounding factors

The motivation for focusing on prior trauma exposure, gender, age, time spent in the country where asylum is applied for and geographical/ethnic orientation is given below.

##### Prior trauma exposure

It is very likely that the population under investigation in this review has been exposed to traumatic pre‐migration events. Pre‐migration trauma exposure is a major determinant for refugee mental health (Kenneth et al., 2011; Ichikawa et al., 2006).

In relation to the expected high pre‐migration trauma exposure, gender and age are important factors to control for.

##### Gender

Women have been found to have higher prevalence rates of PTSD (Naomi et al., 1998; Kessler, 1995). However, this phenomenon can partly be explained by the different types of traumas men and women experience (Pratchett et al., 2010). According to Pratchett et al. (2010), women are more exposed to those types of trauma that are more likely to lead to PTSD symptoms, such as sexual assault. However, gender differences in exposure to different types of trauma cannot fully explain the gender differences in PTSD prevalence (Pratchett et al., 2010; Gavranidou & Rosner, 2003; Halligan & Rachel, 2000), but no other firm explanation for gender differences exists (Halligan & Rachel, 2000). According to Gavranidou and Rosner (2003), the question of whether women are at higher risk of being diagnosed with PTSD is unresolved. Gender (being female) is however found to be a risk factor for other psychiatric disorders (Halligan & Rachel, 2000).

##### Age

Given the different influences on development over the life course, particularly during the early years (Bosquet et al., 2012; Lustig et al., 2003), age is a likely risk factor with respect to the consequences of exposure to trauma.

##### Time since arrival to the country where asylum is applied for

If the non‐detained have stayed for longer in the asylum‐seeking country, they also have had longer time to recover from possible pre‐migration traumas than the detained, and vice versa.

##### Geographical/ethnic orientation

The ways of expressing distress and views of causes differ in some cultures markedly from those of the dominant ‘Western’ culture. Furthermore, although similar symptoms may exist in different cultures, they do not necessarily have the same value or meaning.

##### Unobservables

For the ‘intervention’ under consideration in this review, it is reasonable to expect a certain degree of arbitrariness in the decision process. If the criteria for detention are unclear, this implies that whether or not an asylum seeker is detained is unpredictable. According to the Council of Europe (2010), national detention policies are non‐transparent. Detention of asylum seekers is often applied in a way that is unlawful or arbitrary, and can be arbitrarily prolonged, as, for example, where there is no practical and imminent possibility of removal. In general, detainees have difficulty challenging the legality of their detention (Michael & Liza, 2005; Amaral & Europe, 2010; Council of Europe, 2010).

Although arbitrariness is not randomness, we assessed the degree of arbitrariness in the detention decision process as described by the authors. The risk of systematic differences in unobservable factors between those detained or not detained will probably be minimised if there is a high degree of arbitrariness in the decision process.

##### Effect of primary interest and important co‐interventions

The only effect possible to investigate in this review is the effect of starting and adhering to the intended intervention, that is, the treatment on the treated effect. The risk of bias was therefore assessed in relation to this specific effect.

The risk of bias assessments considered adherence and differences in additional interventions (‘co‐interventions’) between intervention groups. Relevant co‐interventions are those that individuals might receive with or after starting the intervention of interest and that are both related to the intervention received and prognostic for the outcome of interest. Important co‐interventions we considered were any kind of mental health treatments delivered on an individual basis.

##### Assessment

In pairs of two, review authors independently assessed the risk of bias for each relevant outcome from the included studies. We discussed all initial disagreements and were able to reach a consensus in all cases. We report the risk of bias assessment in risk of bias tables for each included study outcome in a supplementary document.

#### 4.3.4 Measures of treatment effect

Reported effect sizes that could not be pooled (were reported in a single study only) were reported in as much detail as possible. For continuous outcomes, effects sizes with 95% confidence intervals (CIs) were calculated using means and standard deviations were available, or alternatively from mean differences, standard errors (SE) and 95% CIs (whichever were available), using the methods suggested by Lipsey and Wilson (2001). Hedges' *g* was used for estimating standardised mean differences (SMD).

For dichotomous outcomes, we calculated odds ratios (ORs) with 95% CIs.

There are statistical approaches available to re‐express dichotomous and continuous data to be pooled together (Sánchez‐Meca et al., 2003). We only transformed dichotomous effect sizes to SMD where appropriate, in the case where one study reported PTSD symptoms as a dichotomous outcome (Forrest & Steel, 2023). To calculate common metric ORs were converted to SMD effect sizes using the Cox transformation.

Software for storing data and statistical analyses were Excel and RevMan 5.4.

#### 4.3.5 Unit of analysis issues

We checked for consistency in the unit of allocation and the unit of analysis, as statistical analysis errors can occur when they are different. There were no studies where the unit of allocation differed from the unit of analysis.

#### 4.3.6 Criteria for determination of independent findings

To account for possible statistical dependencies, we examined a number of issues: whether individuals had undergone multiple interventions, whether there were multiple treatment groups, and whether several studies were based on the same data source.

##### Multiple interventions per individual

There were no studies with multiple interventions per individual.

##### Multiple studies using the same sample of data

Two studies reported on the same group of asylum seekers. In Momartin 2006 (Momartin et al., 2006) and in Steel 2011 (Steel et al., 2011), outcomes were reported on average 3.6 months after release, and Steel 2011 additionally reported outcomes on average 26.3 months after release.

We reviewed both studies, and would only have included one estimate of the effect of detention on average 3.6 months after release. However, neither study was used in the meta‐analysis because the risk of bias was assessed to be too high (see Section 5.2).

##### Multiple time points

Each time point (i.e., currently detained and from the end of detention to 1 year after release) was analysed separately.

#### 4.3.7 Dealing with missing data

Where studies had missing summary data, such as missing standard deviations, we calculated SMDs from mean differences, SE and 95% CIs (whichever were available), using the methods suggested by Lipsey and Wilson (2001).

#### 4.3.8 Assessment of heterogeneity

Heterogeneity amongst primary outcome studies was assessed with the Chi‐squared (*Q*) test, and the *I* ^2^, and *τ* ^2^ statistics (Higgins, 2003). Any interpretation of the Chi‐squared test was made cautiously on account of its low statistical power.

#### 4.3.9 Assessment of reporting biases

Reporting bias refers to both publication bias and selective reporting of outcome data and results. Here, we state how we planned to assess publication bias. We planned to use funnel plots for information about possible publication bias, however we did not find sufficient studies (Higgins & Sally, 2011).

#### 4.3.10 Data synthesis

Meta‐analysis of outcomes was conducted on each metric (as outlined in Section 4.1.4) separately. Studies that were rated critical risk of bias were not included in the data synthesis. The time points of outcome measurement differed between studies. The outcomes at each time point were analysed in separate analyses with other comparable studies taking measures at a similar time point. We grouped outcomes as follows: detained asylum seekers currently detained, from the end of their detention to 1 year after detained asylum seeker's release. None of the studies used in the data synthesis reported outcomes more than a year after release.

We carried out our meta‐analyses using the SMD. All analyses were inverse variance weighted using random effects statistical models that incorporate both the sampling variance and between study variance components into the study level weights. The estimation of *τ* ^2^ was the DerSimonian and Laird (1986) estimate. Random effects weighted mean effect sizes were calculated using 95% CIs, and we provide graphical displays (forest plots) of effect sizes.

#### 4.3.11 Subgroup analysis and investigation of heterogeneity

There were not enough studies to perform moderator analyses.

#### 4.3.12 Sensitivity analysis

There were not enough studies to perform sensitivity analyses.

#### 4.3.13 Treatment of qualitative research

We did not plan to include qualitative research.

#### 4.3.14 Summary of findings and assessment of the certainty of the evidence

‘We did not plan to include Summary of findings and assessment of the certainty of the evidence’.

## 5 RESULTS

### 5.1 Description of studies

#### 5.1.1 Results of the search

The search strategies for the original version of this review were performed between November 2013 and January 2014 and were reported (Filges et al., 2014). The updated search was performed November 2023. We used EPPI Reviewer for screening.

**Figure 1:**
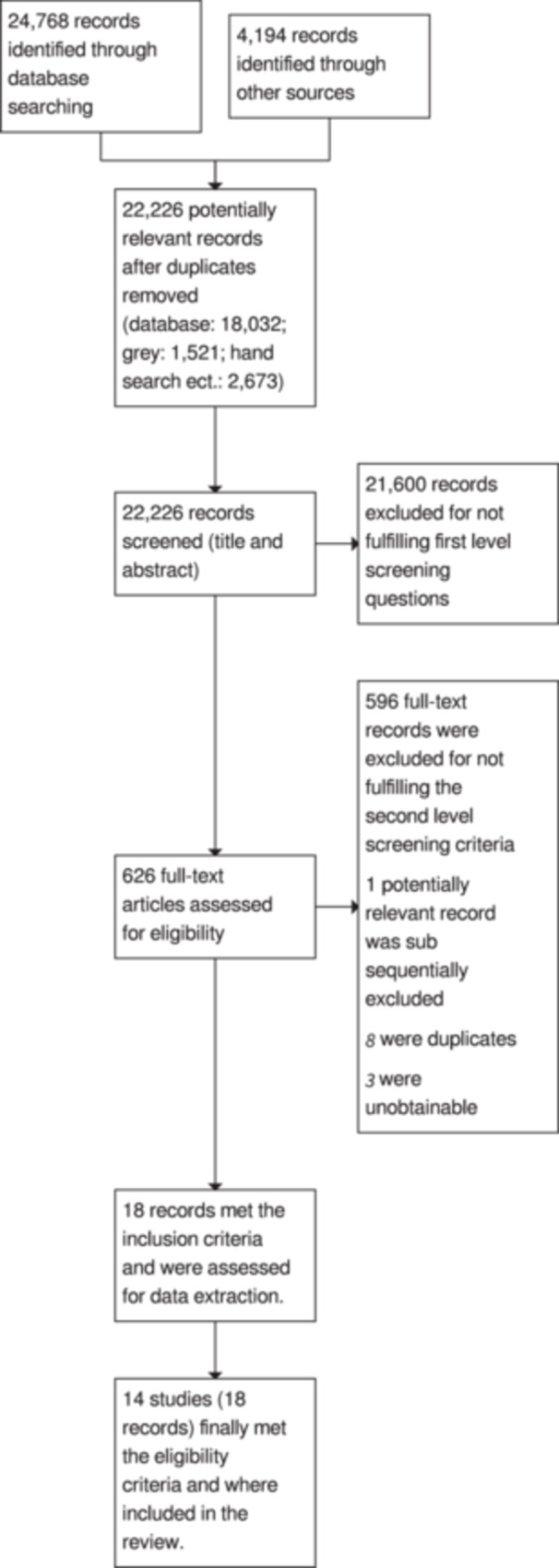
Flow chart.

The results from both searches are summarised in a flow diagram Figure 1. Electronic database searches produced a total of 24,768 records. Of these, 12,218 records were identified in 2012 and 12,550 in 2023. The total number of potentially relevant records was 22,226 after excluding duplicates (database: 18,032; grey: 1521; hand search, snowballing and other resources: 4194). All 22,226 records were screened based on title and abstract; 21,600 were excluded for not fulfilling the first level screening criteria and 626 records were ordered for retrieval and screened in full text. Of these, 596 did not fulfil the second level screening criteria and were excluded. One potentially relevant record was subsequently excluded and 8 were duplicates. Three records were unobtainable despite efforts to locate them through libraries and searches on the internet (Barnes, 1988; Blair, 1996; Fell and Fell, 2010).

Seven records from the snowball search and 5 records from the database searches were included. A total of 14 unique studies, reported in 18 papers, were included in the review.

#### 5.1.2 Included studies

The search resulted in a final selection of 14 studies that met the inclusion criteria for this review. The 14 studies analysed 13 different asylum populations. Two studies, Momartin (2006) and Steel (2011), reported on the same sample of asylum seekers in Australia at different time points after release.

The majority of studies were from Australia (11), one each was from Canada, Japan and the UK.

Prior traumatic experiences are a major determinant for refugee mental health (Carswell et al., 2011; Ichikawa et al., 2006). The population under investigation in this review had experienced a number of traumatic events before fleeing. Seven studies reported a variety of different traumatic events along with the share of asylum seekers having experienced them. Five studies used standard questionnaires to measure the pre‐migration traumatic experiences: section 1 of the Harvard Trauma Questionnaire (HTQ) and Part 1 of the Post‐traumatic diagnostic scale (PDS). Four studies (Cleveland [Cleveland & Rousseau, 2013; Cleveland et al., 2012a, 2012b]; Ichikawa [Ichikawa et al., 2006]; Steel [Steel et al., 2006]; Thompson [Thompson et al., 1998; Silove et al., 1998]) used the HTQ, probably the Indochinese version as they all refer to (Mollica et al., 1992), which describes the development and validation of an Indochinese version of the HTQ which originally included 17 items describing a range of traumatic experiences. In Ichikawa 2006 it is explicitly stated that all 17 original items were included, although only six items were reported. In Cleveland (2013) it is stated that prior trauma was assessed through a 20‐item version of the HTQ Trauma Events Checklist, and all 20 were reported. One study (Robjant et al., 2009; Robjant), used the PDS; 12 different traumas and the share of asylum seekers experiencing them were reported.

In Forrest (2023), six dichotomous indicators of premigration experiences were used and reported as the share of participants reporting ‘yes’ to the indicators and, finally, a testimony method was used in Thompson (2011). The full list of reported traumatic exposures and events is shown in Tables 1 and 2. Further descriptions of all studies are given in the Supporting Information.

**Table 1:** Prior trauma exposures: Treated/comparison.

Study	Thompson (1998)	Ichikawa (2006)^a^	Steel (2006)	Cleveland (2013)
Prior trauma	Percent in treated/comparison group with exposure			
Torture	72/26	67	18/12	43/29
Combat	40/23	80	15/8	27/39
Forced isolation	84/46	80	14/6	43/29
Forced separation from family and friends	‐	80	26/11	65/68
Being close to death	88/40	82	76/29	90/92
Murder of family/friends	92/39	67	75/61	46/53
Witness murder of strangers	96/46	‐	49/32	43/36
Serious injury	‐	‐	14/9	39/35
Imprisonment	‐	‐	37/15	32/21
Mean number of trauma exposures	15/7	9.9/9.5	5.3/3.1	9.3/9.2
Beaten and assaulted	‐	‐	‐	67/76
Family member's health or safety seriously threatened	‐	‐	‐	66/71
Threats or harassment by government or other organised groups	‐	‐	‐	66/64
Family or friends assaulted	‐	‐	‐	60/70
Lack of food or water	‐	‐	46/23	45/41
Unnatural death or disappearance of family or friends	‐	‐	79/62	44/53
Illness without access to medical care	‐	‐	38/16	40/30
Family or friends imprisoned or tortured	‐	‐	‐	39/39
Lack of shelter	‐	‐	19/11	31/24
Kidnapped	‐	‐	11/6	23/17
Brainwashing	‐	‐	13/6	‐
Mean number of trauma exposures^b^	15/7	9.9/9.5	5.3/3.1	9.3/9.2

*Note*: Based on the Harvard Trauma Questionnaire. ‘‐’ means not reported.

^a^ Total sample except mean number of traumatic exposures.

^b^ Not a percent but mean number of exposures.

**Table 2:** Prior trauma exposures: Treated/comparison.

Study	Robjant (2009) (Post‐traumatic diagnostic scale)	Thompson (2011) (A testimony method)	Forrest (2023) (Dichotomous indicators of premigration experiences)^a^
Prior trauma exposure/experiences	Percent in treated/comparison group with exposure		
Torture	39/20	45/68	‐
Combat/war	43/35	21/21	59
Serious physical injury/violence	‐	0/65	33
Nonsexual assault^b^^,^*	46/28	62/47	‐
Sexual assault^c^	21/15	26/33	‐
Imprisonment	43/24	52/12	20
Kidnapped	‐	19/3	‐
Accident/fire/explosion/natural disaster^d^	39/31	5/47	9
Life‐threatening illness	13/17	‐	‐
Threat to life*	‐	93/53	‐
Murder of family/friends*	‐	90/47	‐
Disappearance of family/friends*	‐	88/26	‐
Relative in jail as political prisoner	‐	50/65	‐
Persecution	‐	‐	62
Significant substantial material deprivation	‐	‐	22
Seeing loss of life*	‐	88/68	‐
Witnessed violence in mass demonstrations*	‐	62/23	‐
Search as result of organised violence*	‐	88/59	‐
Forced displacement*	‐	95/6	‐
Lived in refugee camps		5/59	6
Other traumatic event	54/37	‐	‐
Mean number of trauma exposures^e^	2.99/2.17	All are either tortured or have experienced at least two specific traumatic events (marked with *)	‐

*Note*: ‘‐’: not reported.

^a^ Total sample.

^b^ In Robjant (2009) this item is divided into two categories: committed by a known assailant respectively by a stranger. In Forrest (2023) this item is refered to as ‘violence’.

^c^ In Robjant (2009) this item is divided into two categories: committed by a known assailant respectively by a stranger. In Thompson (2011) this item is divided into three categories: Experienced rape, Witnessed rape family (forced within family) and Witnessed rape family (done) respectively.

^d^ In Robjant (2009) this item is divided into two categories: Accident/fire/explosion respectively natural disaster.

^e^ Not a percent but mean number of exposures.

Three studies (Momartin, 2006; Steel, 2011 and Johnston et al., 2009) analysing detained asylum seekers in Australia could not be used in the data synthesis because detention is contaminated with the holding of a Temporary protection visa (TPV). In the studies by Momartin 2006 and Steel 2011 all detained asylum seekers held a TPV, whereas all non‐detained asylum seekers held a Permanent protection visa (PPV). In Johnston (2009), a group of asylum seekers holding a TPV was compared to a group of asylum seekers holding a Permanent humanitarian visa (PHV). Nearly all TPVs (97%) and almost no PHVs (7%) had been held in immigration detention before release into the community (this information was kindly provided by Professor Johnston per e‐mail 12.03 2014). It was not possible to examine for the unique contribution of detention in these three studies. Previous research undertaken with Mandaean Iraqi asylum seekers subject to detention alone or detention and subsequent TPV status has supported a model in which both detention and TPV status were associated with a similar and additive adverse impact on mental health status (Steel, 2011). The studies would therefore most likely seriously overstate the effect of detention on mental health, and they were rated Critical risk of bias on the confounding domain; in accordance with the guidelines for ROBINS I tool we used (Sterne, Hernan, et al., 2016; Sterne, Higgins, et al., 2016), we excluded these from the data synthesis on the basis that they would be more likely to mislead than inform.

In addition, five studies analysing asylum seekers in Australia (Thompson, 1998; Steel, 2006; Thompson, 2011; Rowcliffe et al., 2016 and Mace et al., 2014) were judged to have Critical risk of bias on the confounding domain, and were excluded from the data synthesis on the basis that they would be more likely to mislead than inform.

The remaining six studies, all used in the data synthesis, analysed asylum seekers in the UK (Robjant, 2009), in Japan (Ichikawa, 2006), in Canada (Cleveland, 2013) and Australia (Forrest, 2023, Hedrick et al., 2019 and Zwi et al., 2018).

The main characteristics of the six studies used in the data synthesis are shown in Table 3 and a summary of characteristics are shown in Table 4.

**Table 3:** Characteristics of studies used in data synthesis.

Study	Country	Time period	Sample size (T/C)	Country of origin	Mean age	Share of men	Length of detention	Still detained
Robjant (2009)	UK	Not reported	T:67; C:49	From 43 different countries	29.5 years	60%	Median 1 month	Yes
Ichikawa (2006)	Japan	2002–2003	T: 18; C: 37	Afghanistan	27.8 years	100%	Median 7 months, range is 4–10 months	No
Cleveland (2013)	Canada	2010–2011	T: 122; C: 66	Sub‐Saharan, Middle East and North Africa, South Asia, Latin America, Caribbean and Europe	31.6 years	67%	Mean: 31.2 days	Yes
Forrest (2023)	Australia	2013	T: 193; C: 83	Afghanistan, Iraq, Iran and Pakistan	33.70 years	88%	NR	No
Hedrick (2019)	Australia	2014–2015	T: 3903; C: 23894	NR	NR	87%	NR	Yes
Zwi (2018)	Australia	2014	T: 48; C: 38	Eastern Mediterranean, South East Asian, Western Pacific, African and ‘Stateless’	8.4 years	NR	7 months	Yes

**Table 4:** Summary characteristics of studies used in data synthesis.

Characteristic (Number of comparisons reporting)		
Country (6)	Australia	50%
	UK, Canada, Japan	50%
Time period (5)	Median	2013
	Range	2002–2014
Number of participants, detained (6)	Median	95
	Range	18–3903
Number of participants, control (6)	Median	58
	Range	37–23,894
Percent male (5)	Median	87%
	Range	60%–100%
Age (5)	Median	29.5
	Range	8.4–33.7
Length of detention (4)	Median	4 months
	Range	1–7 months
Still detained (6)	Yes	67%

The reported time period spanned by the included studies is 10 years, from 2002 to 2015. In four studies, the asylum seekers originated from a variety of countries; in one study the common country of origin was Afghanistan; and in one study the countries of origin were not reported. In total, 27,797 asylum seekers were analysed, of which 14% had been detained. The median sample size of detained asylum seekers was 95 with a range of 18 to 3903. The median sample size of non‐detained asylum seekers was 58 with a range of 37 to 23,894. The mean age of the detained asylum seekers varied between 8.4 years and 33.7 years. In all studies, men accounted for more than 50% of the sample. The measure of length of detention varied between studies, with two reporting median length and two reporting mean length. Two studies did not report the length of detention. In the four studies reporting detention length, the reported median or mean lengths of detention was less than a year; however, in three of these studies the asylum seekers were still detained at the time of interviewing.

##### Characteristics of detention centres

Two of the studies provided general information about detention practices and on the characteristics of detention centres in the countries in question.

For Canada, Cleveland (2013) provided general information about living conditions in Canadian detention centres. The detention centres are prisons, men and women are held in separate wings, there are virtually no activities and only primary health care is provided.

Robjant (2009) provided information about the detention centres and living conditions from which participants were recruited in the UK. Two of the centres were high security centres with a large number of former male prisoners. The other two centres held male and female detainees, and also each had a family wing and hence detained children of any age, with their parents. Various activities were available and healthcare was provided on site and was privately run.

Unfortunately, the study from Japan, Ichikawa (2006), provided no information on detention centres and living conditions in Japan.

The Australian studies (Forrest, 2023; Hedrick, 2019; Zwi, 2018) did not provide much information on the characteristics of detention centres.

According to Forrest (2023): ‘Under the Migration Act, any noncitizen found in Australia without a valid visa must be detained, irrespective of their individual circumstances' (Migration [Australia] Act 1958, s.189). Thus, anyone who attempts to enter Australia without valid authorisation is subject to automatic detention’ (Forrest 2023, p. 643). Other than that, nothing was reported except the detained asylum seekers analysed were all held in detention centres inside Australia, that is, they were not deported to Nauru or Papua New Guinea.

In Hedrick (2019), three types of detention facilities were examined; onshore detention, offshore detention (Nauru), and offshore detention (Manus Island). Onshore immigration detention includes centres on the Australian mainland as well as on Christmas Island, a remote island located in the Indian Ocean. In the onshore detention network, asylum seekers are detained in both high‐security immigration detention facilities (with razor wire fences, surveillance, and other prison‐like features and practices), and low‐security accommodation (with a more domestic environment than other forms of detention, often used for families with children).

The characteristics of offshore processing (outsourced to private contractors by the Australian government, and referred to as ‘regional processing’) on Nauru and Manus Island have garnered a lot of attention. The Nauru Regional Processing Centre is an offshore Australian immigration detention facility in use since 2001 (Karlsen, 2016). It is located on the South Pacific island nation of Nauru and run by the government of Nauru. The Nauru facility was opened in 2001 as part of the Howard government's Pacific Solution (Phillips, 2012).

The Manus Regional Processing Centre, or Manus Island Regional Processing Centre (MIRCP), was one of a number of offshore Australian immigration detention facilities (Karlsen, 2016). The centre was located on the PNG Navy Base Lombrum on Los Negros Island in Manus Province, Papua New Guinea. It was originally established in 2001, along with the Nauru Regional Processing Centre, as an ‘offshore processing centre’.

Four of the six studies used in the data synthesis reported on prior traumatic exposures. The 12 most reported prior traumatic exposures along with the mean number of trauma exposures is shown in Table 5.

**Table 5:** Percent reporting prior traumatic experiences in studies used in data synthesis.

Prior trauma	Ichikawa (2006)	Cleveland (2013)	Robjant (2009)	Forrest (2023)
Torture	67	43	39	‐
Combat/war	80	27	43	59
Forced isolation	80	43	‐	‐
Forced separation from family and friends	80	65	‐	‐
Being close to death	82	90	‐	‐
Murder of family/friends	67	46	‐	‐
Witness murder of strangers	‐	43	‐	‐
Serious injury/violence	‐	39	13	33
Imprisonment	‐	32	43	20
Persecution	‐	‐	‐	62
Mean number of traumatic experiences	10	9	3	‐

*Note*: ‘‐’ means not reported.

In three out of four studies reporting on traumatic events, 39% to 67% of the detained asylum seekers had experienced torture. Combat/war, murder of family and friends, forced isolation, serious injury/violence, persecution and imprisonment have also been commonly experienced amongst the detained asylum seekers.

#### 5.1.3 Excluded studies

In addition to the 14 studies that met the inclusion criteria for this review, two studies (Essex et al., 2022; Keller et al., 2003) at first sight appeared relevant but did not meet our criteria. The studies and reason for exclusion are given in Table 6.

**Table 6:** Studies excluded with reason.

Study	Reason for exclusion
Keller (2003)	The study analysed detained asylum seekers in the USA. The comparison group was released detained asylum seekers. Hence, it did not qualify for inclusion in the review.
Essex (2022)	All detained for various length of time

### 5.2 Risk of bias in included studies

The risk of bias coding for each of the 14 studies and their outcomes is shown in Supporting Information.

All studies used non‐randomised designs, and were rated using the ROBINS‐I tool.

Nine studies used opportunity sampling strategies and two studies in addition relied on snowball sampling. A detailed description of the sampling techniques is given in Table 7.

**Table 7:** Sampling techniques.

Study	Sampling techniques
Cleveland (2013)	Opportunity sampling: For the adult study, we interviewed 122 adult asylum seekers detained (at least 7 days) in either the Laval (Montreal) or the Toronto Immigration Holding Centre. A comparison group of 66 recently‐arrived (within a year) adult asylum seekers who had never been detained in Canada completed the same questionnaires. For both the detained and nondetained groups, the study sample is highly representative. For the detained sample, researchers visited the Laval and Toronto Immigration holding Centres weekly in 2010–2011 and invited all asylum seekers who had been detained for at least a week to take part in the study. The nondetained sample was recruited through community and government agencies providing residential and settlement services to asylum seekers in Montreal and Toronto. Researchers did not select or filter participants in any way. All eligible individuals, without distinction, were invited to participate.
Forrest (2023)	Used the Longitudinal Study of Humanitarian Migrants (BNLA), a national longitudinal survey of recently arrived refugees who were granted asylum in Australia. All offshore applicants, including both primary and secondary applicants, who arrived in Australia between May and December 2013, and all onshore migrants who were granted permanent protection in the same period were eligible to participate in the survey. The survey sample was selected at random from the Australian Government's settlement database. For the current study the sample used was restricted to onshore migrants who were listed as the primary applicant on their visa application (*N* = 334).
Hedrick (2019)	Self‐harm incident reports were obtained from the Department of Immigration and Border Protection. The reports contain all self‐harm incidents among the whole Australian asylum seeker population that detention and community‐based staff and contractors are required to report. The reports are refering to self‐harm incidents occurring between 1st August 2014 and 31st July 2015.
Ichikawa (2006)	Opportunity sampling: contacted them through their lawyers or non‐governmental organisations. Of 73 contacted, 55 agreed to participate.
Johnston (2009)	Opportunity sampling and snowballing targeting women and men of varying ages, educational backgrounds and family compositions (e.g., intact and nonintact nuclear families). Excluded if they had not been living at least 6 months in the community (outside detention) and could not speak Arabic or English. Participants were recruited through community organisations such as Migrant Resource Centres and non‐government organisations providing services to refugees in the study site. Community health centres were not included as points of contact to avoid over‐representation of ‘patients’. Refugees who did not utilise these community services were accessed by snowballing within established community networks.
Mace (2014)	Used data from the revised health‐screening questionnaire used for all new patients reviewed by Princess Margaret Hospital (PMH) Refugee Health Service (RHS). The cohort studied was comprised of school‐aged children (4–18 years old) with a completed pro forma. Excluded were children in active/guarded detention.
Momartin (2006)/Steel (2011)	Opportunity sampling: The sample was recruited consecutively from the Early Intervention Programme of the Service for the Treatment and Rehabilitation of Torture and Trauma Survivors (STARTTS) in Sydney, New South Wales. Resettlement agencies in NSW are required to refer recent refugees (both TPV and PPV holders) to the programme, irrespective of their mental status or level of exposure to past trauma.
Robjant (2009)	Opportunity sampling: Treated: From four centres, recruited from the library and other communal areas, 75% agreed, main reason for not participating was language problems; Comparison: recruited from seven different community drop in centres, 60% of those approached agreed to participate.
Rowcliffe (2016)	Used clinical data recorded during standardised assessment of new patients referred to the Princess Margaret Hospital Refugee Health Service between October 2013 and December 2014.
Steel (2006)	Opportunity and snowball sampling: Lists of names provided by community leaders were supplemented by snowball sampling to recruit 241 Arabic‐speaking Mandaean (from Iraq or Iran)refugees in Sydney (60% of the total adult Mandaean population).
Thompson (1998)	Opportunity sampling: Comparison: Information about the study was provided through legal aid and resettlement services, ethnic radio stations, newspapers, newsletters, magazines and community meetings. It was emphasised that the research team was independent of any government department, and anonymity of responses was assured. All adult Tamils were invited to participate, irrespective of their residency status. Legal agencies in contact with asylum‐seekers and the Ealam Tamil Association agreed to mail questionnaires to their clients or membership without revealing individuals' names to the researchers. The Ealam Tamil Association provides a focus for cultural and social support for the Tamil community, and its membership is not limited to any particular sector or political faction (Silove et al., 1998). Treated: Tamils from Sri Lanka detained in the Maribyrnong Detention Centre.
Thompson (2011)	Selective opportunity sampling: Different ethnic organisations, the divisions of general medical practices, as well as legal agencies working with asylum seekers living in the community or in detention, were involved in asking their clients if they would participate in the research. All participants from an immigration detention centre who were seeking asylum were invited to participate in the study. The final included sample was selected based upon being either a survivor of torture of a survivor of other types of systemic abuse.
Zwi (2018)	Opportunity sampling: The community sample (aged 6 months to 15 years) was recruited from a population cohort of all newly arrived children settled in a non‐urban area. They were visited by nurses at their home between 2009 and 2013 and their families were invited to participate in the study. Data for detained children were collected in March 2014 during the Australian Human Rights Commission (AHRC) National Inquiry into Children in Immigration Detention.

Table 8 shows a summary of the risk of bias associated with the studies. One study was rated differently on different outcomes, the most favourable rating is included in the summary risk of bias table. We stopped the assessment of a non‐randomised study outcome when it was rated ‘Critical’ on any of the items. Therefore, not all studies are rated on all domains.

**Table 8:** Risk of bias summary.

Judgement:	Low risk of bias	Moderate risk of bias	Serious risk of bias	Critical risk of bias	No information	Not rated on this domain
Risk of bias domain						
Overall Judgement	0	4	2	8	0	0
Confounding bias	2	2	2	8	0	0
Selection of participants	4	2	0	0	0	8
Classification of intervention status	5	1	0	0	0	8
Deviation from intervention	6	0	0	0	0	8
Missing Outcome Data	4	1	0	0	1	8
Measurement of Outcome	0	5	1	0	0	8
Selection of Reported Results	1	5	0	0	0	8

*Note*: We stopped the assessment of a non‐randomised study outcome when it was rated ‘Critical’ on any of the items. Therefore, not all studies are rated on all domains.

Eight studies were rated Critical risk of bias on the Overall judgement item, corresponding to a risk of bias so high that the findings should not be considered in the data synthesis. The overall Critical risk of bias rating was due to issues on the Confounding bias item; all eight were rated Critical risk of bias on this item; that is, they failed to establish a comparison group that was balanced on important confounders and further did not control for any confounders.

In three studies (Johnston, 2009; Momartin, 2006; Steel, 2011) all (almost all in Johnston 2009) detained asylum seekers were also holders of a TPV and were compared to non‐detained holders of a PPV. In addition, three studies (Steel, 2006; Thompson, 1998, Thompson, 2011) did not adjust for confounding and there were some large imbalances on important confounders. Two studies did not consider any confounders at all (Mace, 2014; Rowcliffe, 2016); they were therefore rated to have a Critical risk of bias on the confounding item.

Two studies were rated Serious risk of bias overall and four studies were rated Moderate risk of bias overall.

Of the six studies not rated Critical risk of bias overall, two studies had serious issues on the Confounding item, two had Moderate issues and two were rated Low risk of bias. On the Selection bias item, four were rated Low risk of bias and two were rated Moderate risk of bias. Five studies were rated Low risk of bias on the Classification item and one was rated Moderate risk of bias; all six were rated Low risk of bias on the Deviation item. One study did not provide enough information to be rated on the Missing data item, whereas four were rated Low risk of bias and one was rated Moderate risk of bias. On the Measurement item, five were rated Moderate risk of bias and one was rated Serious risk of bias. Five studies were rated Moderate risk of bias on the Selection of Reported Results mainly because there was no a priori analysis plan and one study had a published protocol and no other issues and was rated Low risk of bias.

### 5.3 Synthesis of results

Of the 14 included studies, eight were judged to have a critical risk of bias and thus were not included in any syntheses. Of the six studies that did not have critical risks of bias, all studies provided data enabling the calculation of either a SMDs or ORs and their SE. Four studies reported outcomes while the detained asylum seekers were still detained, and two studies reported outcomes less than 2 years after release of the detained asylum seekers.

#### 5.3.1 Mental health outcome results

The mental health outcomes measures reported in the studies were PTSD, depression, anxiety, social–emotional wellbeing, nonspecific psychological distress, and self‐harm. PTSD, depression, anxiety and social–emotional wellbeing were assessed using standardised measures. PTSD was assessed using the HTQ and the Impact of Events Scale‐revised (IES‐R). Depression and anxiety were assessed using the Hopkins Symptoms Checklist‐25 (HSCL‐25) and the Hospital Anxiety and Depression scale (HADS (D and A). Social–emotional wellbeing, assessed using the parent version of the strength and difficulties questionnaire (SDQ). Nonspecific psychological distress was assessed using the Kessler–6 Psychological Distress Scale (K6). Self‐harm incidents were recorded by detention and community‐based staff and contractors as required by contractual arrangements between the Department of Immigration and Border Protection (DIBP) and the immigration detention and community‐based service providers. The incident reports are sent to the DIBP where they are archived in a centralised database (Commonwealth Immigration Ombudsman, 2024). Eleven different types of self‐harm methods were used by the Australian asylum seeker population during the study period. The five most commonly used methods were cutting, self‐battery (defined as striking one's body against hard objects or beating oneself heavily and repeatedly to cause injury), hanging, self‐poisoning by medication and self‐poisoning by chemicals.

No other mental health outcomes were reported in the studies used in the data synthesis.

All outcomes are measured such that a negative effect size favours the detained asylum seekers, that is, when an effect size is negative, the detained asylum seekers are better off than the comparison groups of non‐detained asylum seekers, and when an effect size is positive, the non‐detained asylum seekers are better off than the detained asylum seekers.

##### PTSD

Two studies reported PTSD while detained asylum seekers were still detained and two other studies reported PTSD after their release from detention.

###### Detained asylum seekers still detained

**Figure 2:**
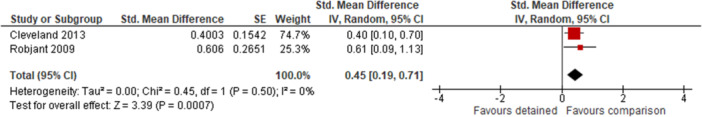
Forest plot PTSD while in detention.

There was no heterogeneity between the two studies reporting PTSD while the asylum seekers were still detained; the estimated *τ* ^2^ is 0.00 and *I* ^2^ is 0% as displayed in Figure 2. Both effect sizes favour the comparison group and are statistically significant. The weighted average SMD is 0.45 [95% CI 0.19, 0.71].

###### After release of detained asylum seekers

**Figure 3:**
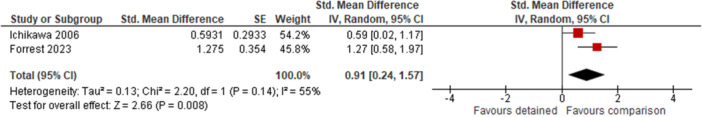
PTSD follow‐up.

There is some heterogeneity between the two studies reporting PTSD after release; the estimated *τ* ^2^ is 0.13 and *I* ^2^ is 55% as displayed in Figure 3. The pooled estimate and CI should therefore be interpreted with caution. Both effect sizes favour the comparison group and are statistically significant. The weighted SMD is 0.91 [95% CI 0.24, 1.57].

##### Depression

Two studies reported depression while still detained and one other study reported depression after release from detention.

###### Detained asylum seekers still detained

**Figure 4:**
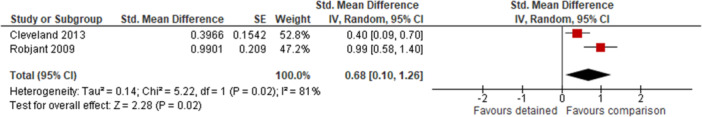
Depression while in detention.

There is some heterogeneity between the two studies reporting depression while the asylum seekers are still detained; the estimated *τ* ^2^ is 0.14 and *I* ^2^ is 81% as displayed in Figure 4. The pooled estimate and CI should therefore be interpreted with caution. Both effect sizes favour the comparison group and are statistically significant. The weighted average SMD is 0.68 [95% CI 0.10, 1.26].

###### After release of detained asylum seekers

**Figure 5:**
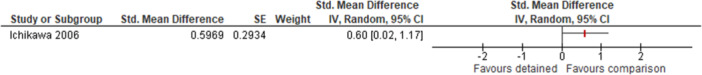
Depression follow‐up.

The effect size after release favours the comparison group and is statistically significant. Ichikawa reports a SMD of 0.60 [95% CI 0.02, 1.17] less than a year after release as displayed in Figure 5.

##### Anxiety

Two studies reported anxiety while still detained and one other study reported anxiety after release from detention.

###### Detained asylum seekers still detained

**Figure 6:**
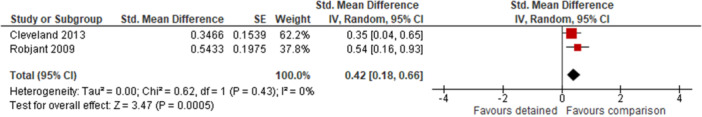
Anxiety while in detention.

There is no heterogeneity between the two studies reporting anxiety while the asylum seekers are still detained; the estimated *τ* ^2^ is 0.00 and *I* ^2^ is 0% as displayed in Figure 6. Both effect sizes favour the comparison group and are statistically significant. The weighted average SMD is 0.42 [95% CI 0.18, 0.66].

###### After release of detained asylum seekers

**Figure 7:**
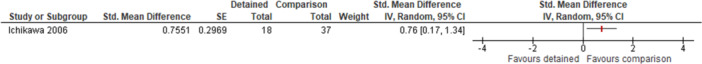
Anxiety follow‐up.

The effect size after release favours the comparison group and is statistically significant. Ichikawa reports a SMD of 0.76 [95% CI 0.17, 1.34] less than a year after release as displayed in Figure 7.

##### Nonspecific psychological distress

No studies reported psychological distress while detained and one study reported after release.

###### After release of detained asylum seekers

**Figure 8:**
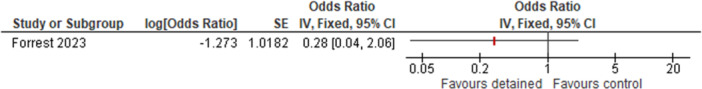
Nonspecific psychological distress.

The effect size favours the detained group and is not statistically significant; an OR of 0.28 [95% CI 0.04, 2.06] is reported as displayed in Figure 8.

##### Self‐harm

One study reported incidents of self‐harm (excluding suicide) while detained asylum seekers were still detained and none after release.

###### Detained asylum seekers still detained

**Figure 9:**
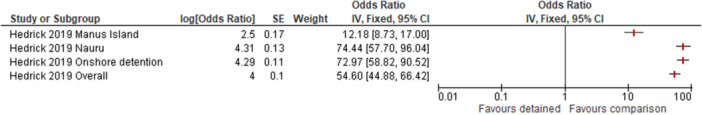
Self‐harm while detained.

Incidents were reported separately for the three types of detention: Manus Island, Nauru and onshore detention. We calculated the OR for each type of detention and an overall OR as well. All effect sizes favour the comparison group, are statistically significant and very high, as displayed in Figure 9.

For asylum seekers living in detention on Manus Island, the OR was 12.18 [95% CI 8.73, 17.00]. For asylum seekers living in detention in Nauru, the OR was 74.44 [95% CI 57.70, 96.04]. For asylum seekers living in onshore‐detention, the OR was 72.97 [95% CI 58.82, 90.52]. Overall, the OR for asylum seekers living in detention was 54.60 [95% CI 44.88, 66.42].

##### Social–emotional wellbeing

One study reported social‐emotional well‐being while detained asylum seekers were still detained and none reported after their release.

###### Detained asylum seekers still detained

One study reported social‐emotional well‐being for a sample of children held in detention on Christmas Island (a remote Indian Ocean island) aged 4–15 years.

**Figure 10:**
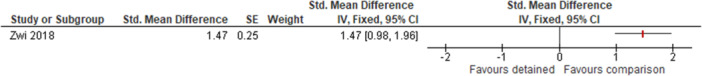
Social‐emotional well‐being.

The effect size favours the comparison group and is statistically significant; a SMD of 1.47 [95% CI 0.98, 1.96] is reported as displayed in Figure 10.

## 6 DISCUSSION

### 6.1 Summary of main results

The studies used in the data synthesis reported outcomes on mental health, measured as PTSD, depression, anxiety, psychological distress, self‐harm and social functioning.

Primary study effect sizes for PTSD, depression and anxiety while the asylum seekers were still detained lies in the range 0.35 to 0.99, all favouring the non‐detained asylum seekers.

The weighted average effect sizes while detained for PTSD and anxiety are of a magnitude which may be characterised as being of clinical importance and the weighted average effect size for depression is of an even higher magnitude. They all favour the non‐detained, that is, there is an adverse effect of detention on mental health. The magnitude of the pooled estimates should, however, be interpreted with caution as they are based on two studies (Cleveland, 2013; Robjant, 2009), and for depression there is some inconsistency in the magnitude of effect sizes between the two studies, one effect size is moderate (0.4) and the other is large (0.99).

Two studies (Forrest, 2023; Ichikawa, 2006) reported PTSD after release and the weighted average effect size is even higher than while detained with primary study effect sizes of 0.59 and 1.27.

One study (Ichikawa, 2006) reported the outcomes of depression and anxiety after release; the effect sizes are all of clinical importance and favour the non‐detained asylum seekers.

One study each (Forrest, 2023; Hedrick, 2019; Zwi, 2018) reported outcomes related to psychological distress, self‐harm and social functioning. Psychological distress favoured the detained but was not statistically significant; whereas both the studies reporting on self‐harm and social functioning reported high negative impacts of detention; in particular, the effect sizes of self‐harm were extremely high.

### 6.2 Overall completeness and applicability of evidence

In this review we included six studies in the data synthesis. This number is relatively low compared to the number of studies (14) meeting the inclusion criteria. The reduction was caused by two different factors. Unfortunately, three studies (of which one was a follow‐up to another) compared detained asylum seekers holding TPVs to non‐detained asylum seekers holding PPVs or PHVs (Johnston, 2009; Momartin, 2006; Steel, 2011). It was not possible to examine for the unique contribution of detention in these studies. They were rated critical risk of bias in the confounding domain and, in accordance with the protocol, were not used in the data synthesis. Almost all studies (two exceptions) collected information on some or all of the pre‐specified confounding variables (see the supplementary document). Unfortunately, three studies (Steel, 2006; Thompson, 1998, 2011) did not adjust for confounding and there were some large imbalances on important confounders. They were rated critical risk of bias in the confounding domain and, in accordance with the protocol, we excluded these from the data synthesis on the basis that they would be more likely to mislead than inform. Two studies did not consider any confounders at all (Mace, 2014; Rowcliffe, 2016).

A larger number of useable studies in the data synthesis would have provided a more robust literature on which to base conclusions.

One study used the entire population of asylum seekers in Australia between 1st August 2014 and 31st July 2015. The remaining studies used opportunity sampling strategies (two studies in addition relied on snowball sampling). The populations under investigation in the included studies may therefore, with one exception, not be representative of the general population of detained asylum seekers.

Studies investigating asylum seekers detained in four different countries (Australia, Canada, UK and Japan) were identified, and the asylum seekers originated from a variety of countries. However, none of the six studies investigating detention of asylum seekers in Australia were used in the data synthesis for the reasons given above. This is a clear limitation of the review as Australia has been unique in establishing a policy of mandatory detention of all asylum seekers arriving by boat or without valid travel documents.

### 6.3 Quality of the evidence

All studies used non‐randomised designs, thus we are aware that strong causal conclusions cannot be drawn from the included studies.

Considering the particular population under investigation in this review, it is essential that an appropriate comparison group is used to establish causality. All studies that were included used asylum seekers not detained as a comparison, which is a precondition for being an appropriate comparison group.

The quality of the evidence in this review was enhanced by excluding studies assessed to be at critical risk of bias using the ROBINS–I tool from the data synthesis. We believe this process excluded those studies that are more likely to mislead than inform.

Due to the sampling strategies used in all but one study (opportunity sampling and snowball sampling), obtaining balance on the confounding factors may be difficult and probably requires some luck.

Nevertheless, four of the six studies used in the data synthesis had no large imbalances on the pre‐specified confounders, and three of these studies in addition statistically controlled for the confounders.

Risk of bias due to confounding was rated not to be of concern in two studies, of some concern in two studies and of serious concern in two studies.

There was overall consistency in the direction of treatment effects in that all treatment effects favoured the non‐detained. For depression, while still detained, there is, however, some inconsistency in the magnitude of effect sizes between the two studies included in the analysis.

The magnitude of all pooled estimates in this review should be interpreted with caution as they are based on two studies.

### 6.4 Potential biases in the review process

We performed a comprehensive electronic database search, combined with grey literature searching, and citation screening of relevant studies and reviews. All citations were screened in teams by two independent review authors and one research assistant (TF, MWK, FMBG).

We believe that all the publicly available studies on the effect of detaining asylum seekers on their mental health, physical health and social functioning up to the censor date were identified during the review process.

However, three references were not obtained in full text (Barnes, 1988; Blair, 1996; Fell, 2010). A potential for bias arises from omitting these three unobtainable studies.

We are unable to comment on the possibility of publication bias as at most two comparisons were included in each meta‐analysis.

We believe that there are no other potential biases in the review process as two review authors and one research assistant in pairs of two (TF, MWK and FMBG) independently coded the included studies. Any disagreements were resolved by discussion. Assessment of study quality and numeric data extraction was made by one review author (TF) and each study was checked by one other review author (MWK). There were only minor disagreements and they were resolved by discussion.

### 6.5 Agreements and disagreements with other studies or reviews

We identified three systematic reviews on the mental health impacts of detention of asylum seekers (Robjant et al., 2009; Tania & Marianne, 2013) including an update (von Werthern et al., 2018; Mares, 2021). All reviews provided a narrative synthesis.

In Tania and Marianne (2013), the primary aim was to study the impact of detention of torture survivors, although primary studies where only some participants were torture survivors were also included. The author's conclusion is that although the studies do report severe mental health issues amongst detained torture survivors and, in general, serious mental health problems are found, the available data is insufficient to allow analysis of any specific effects.

The review by Robjant et al. (2009) included all studies that reported quantitative or qualitative measures of mental health for children, adolescents or adults who were either currently detained or who had previously been detained in immigration detention or removal centres in Australia, the UK or the USA. The authors concluded that primary studies consistently report high levels of mental health problems amongst detainees and there is some evidence to suggest an independent adverse effect of detention on mental health. However, they also note that research on this topic is in its infancy and primary studies are limited by methodological constraints. The review was updated in 2018 (von Werthern et al., 2018). The updated search did not place any restrictions on country of detention. The updated review provides a narrative synthesis which supports the findings from the 2009 review.

In Mares (2021) a scoping review was performed to answer the question: ‘What is the current evidence in the peer reviewed literature, about the impact of immigration detention on children and families who seek asylum?’ (Mares, 2021). included all studies that reported mental health and/or developmental outcomes of currently or former detained populations of children, adolescents and/or families who were refugees or seeking asylum. Based on a narrative analysis, the authors conclude that there are high rates of distress, mental disorder, physical health and developmental problems in children aged from infancy to adolescence which persist after resettlement. Restrictive detention is a particularly adverse reception experience and children and parents should not be detained or separated for immigration purposes. In line with Robjant et al. (2009) and the update (von Werthern et al., 2018) they also note that research on this topic is limited and primary studies all have acknowledged methodological weaknesses.

The three reviews and the update focus on different populations to the one in our review, have limitations of different kinds (limited to torture survivors in Tania & Marianne, 2013, limited to Australia, the UK or the USA in Robjant et al., 2009) and limited to children and adolescents in Mares (2021), do not apply restrictions to the quality of studies and none perform meta‐analyses but rely on a narrative synthesis. In our review, no limitations of this kind are employed, and we perform a meta‐analysis where possible.

Consistent with our conclusions, though, the apparent feedback from all three reviews and our update is that more research is needed. In addition, Robjant et al. (2009) and the update (von Werthern et al., 2018) conclude that the current evidence suggests an independent adverse effect of detention on mental health, which is in line with our conclusion.

## 7 AUTHORS' CONCLUSIONS

### 7.1 Implications for practice and policy

The process of seeking asylum in Western countries places additional demands on asylum seekers. These include, besides detention, enforced dispersal within the community, more stringent refugee determination procedures, and temporary forms of asylum. In several countries, asylum seekers living in the community face restricted access to work, education, housing, welfare and, in some situations, to basic health care services. Thus, post‐migratory stressors of various kinds seem to negatively affect this population who are already vulnerable to mental health difficulties as a result of their previous exposure to traumatic events.

Considering the fact that the population under investigation in this review has high rates of pre‐migration trauma and that detention is not the only post‐migration stressor, it was essential that an appropriate comparison group was used to establish causality.

All studies included in the data synthesis compared detained asylum seekers with a group of asylum seekers living in the community who had experienced similar traumatic events before arrival. Despite facing comparable post‐migration adversities and prior traumatic exposure, all studies reported adverse effects on the mental health of detained asylum seekers. There is thus some evidence to suggest an independent deterioration of the mental health due to detention of a group of people who are already highly traumatised.

Furthermore, adverse effects on mental health were found not only while the asylum seekers were detained, but persisted beyond the period of detention, as evidenced by two studies analysing asylum seekers post‐release. This suggests that the adverse mental health effect of detention may be prolonged, extending well beyond the point of release into the community.

Though, based on a single study, we find it important to mention the effects sizes showing alarming high odds of self‐harm for detained asylum seekers in Australia. Compared to community‐based asylum seekers, Hedrick (2019) reports an OR for asylum seekers living in detention overall of 54.60 and statistically significant. The OR of self‐harm was further reported separately for asylum seekers detained in three types of detention: Manus Island, Nauru and onshore detention. The ORs were in the range 12.18 to 74.44; all were significant.

As stated by Hedrick (2019) in his conclusion: ‘These findings clearly illuminate the deleterious impact of immigration detention on the health of detained asylum seekers; the extremely high self‐harm rates identified in the present study are cause for considerable concern and warrant urgent attention’.

Considering the potential adverse effects of detention on the mental health of already traumatised asylum seekers, the use of detention should be discontinued altogether or reserved strictly as a last resort, justified by purposes beyond the mere status of being an asylum seeker.

The necessity of exploring and implementing alternatives to immigration detention is firmly entrenched within both European and international legal frameworks. In recent years, there has been increasing focus on how these alternatives can assist states in managing migration without excessively resorting to depriving individuals of their freedom.

The Council of Europe (2019) suggest a range of different alternatives, including registration with authorities; temporary authorisation; case management or case‐worker support; family‐based care (for unaccompanied children and/or separated children); residential facilities; open or semi‐open centres; regular reporting; designated residence; supervision; return counselling; return houses; bail, bond guarantor or surety; or electronic monitoring.

Many of these alternatives, however, restrict the movement or deprive the liberty of asylum seekers and are thus subject to human rights oversight. The type of alternative to detention that a government uses must fit the country's particular context, and especially the needs of the individual asylum seeker. The least intrusive alternative must always be taken in each individual case.

The Council of Europe identifies ‘essential elements’ of effective implementation of the alternative. These elements are: screening and assessment; access to information; legal assistance; case management services; dignity and human rights; and trust in asylum and migration procedures.

These elements should be taken into consideration when implementing alternatives to detention.

### 7.2 Implications for research

Further research is required to fully address the potential adverse effects on the mental health of detained asylum seekers. Few studies have investigated this issue using appropriate comparison groups, and even fewer studies have investigated the long‐term effects after release.

It should be acknowledged that research in this field is problematic for a number of practical and methodological reasons. Researchers report encountering difficulties in acquiring access to detained asylum seekers. The small sample sizes recruited for some of the studies probably reflect some of these practical difficulties. However, sampling methods targeting individuals who have been released from detention at the time of the study, allows investigation of the longer‐term impact of detention.

Due to the nature of the research field, future studies will probably have to rely on opportunity sampling strategies and/or snowball sampling, as did most of the studies in this review. Obtaining balance on important confounding factors may be difficult, which adds to the importance of statistically controlling for relevant factors.

A few of the studies report only descriptive results even though data had been gathered on important confounding factors, such as prior traumatic experiences. The risk of bias due to confounding would be judged to be of less concern had the primary study authors controlled for these factors. As the data already are gathered it is recommended that analyses controlling for important confounding factors are carried out using these data.

Although the six studies used in the data synthesis cover people seeking asylum in four different countries, research from more countries is needed to generalise the results as conditions of detention varies across countries. As we recommend that the use of detention should in general come to an end or at least be used only as an absolutely last resort, these future studies will probably have to rely on sampling methods targeting individuals who have experienced detention but have been released at the time of the study, allowing investigation of only the longer‐term impact of detention.

## CONTRIBUTIONS OF AUTHORS

Please give brief description of content and methodological expertise within the review team. The recommended optimal review team composition includes at least one person on the review team who has content expertise, at least one person who has methodological expertise and at least one person who has statistical expertise. It is also recommended to have one person with information retrieval expertise.

Who is responsible for the below areas? Please list their names:

- Content: Trine Filges, Edith Montgomery
- Systematic review methods: Trine Filges, Malene Wallack Kildemoes
- Statistical analysis: Trine Filges
- Information retrieval: Elizabeth Bengtsen

## DECLARATIONS OF INTEREST

Trine Filges and Edith Montgomery authored the prior published review on the topic.

### Preliminary timeframe

This is an update of a prior review.

### Plans for updating this review

Trine Filges will be responsible for future updates of the review.

## SOURCES OF SUPPORT

### Internal sources

- VIVE Campbell, Denmark.

### External sources

- None, Other.

## REGISTRATION AND PROTOCOL

Filges et al. (2014).

## DATA, CODE AND OTHER MATERIALS

Supporting Information: [Link], [Link], [Link], [Link], [Link].

### PEER REVIEW

The peer review history for this article is available at https://www.webofscience.com/api/gateway/wos/peer-review/10.1002/cl2.1420.

## Supporting information

Supporting information.

Supporting information.

Supporting information.

Supporting information.

Supporting information.

## References

[cl21420-bib-0001] Abernathy, B. E. (2008). Who am I now? Helping trauma clients find meaning, wisdom, and a renewed sense of self. In G. R. Walz , J. C. Bleuer , & R. K. Yep (Eds.), Compelling counseling interventions (pp. 199–208). Counseling Outfitters, LLC.

[cl21420-bib-0002] Amaral, P. , & Jesuit Refugee Service Europe . (2010). Becoming vulnerable in detention: Civil society report on the detention of vulnerable asylum seekers and irregular migrants in the European Union (The DEVAS Project). Jesuit Refugee Service (Europe).

[cl21420-bib-0003] American Civil Liberties Union (ACLU) . (2007). Briefing materials submitted to the United Nations special rapporteur on the human rights of migrants. Detention and Deportation Working Group. http://www.aclu.org/pdfs/humanrights/detention_deportation_briefing.pdf

[cl21420-bib-0004] Barnes, D. M. (1988). Policies, programs and outcomes for unaccompanied Vietnamese refugee minors in Australia. University of New South Wales (Australia).

[cl21420-bib-0005] Blair, R. G. (1996). Risk and protective factors in the mental health status of Cambodian refugees in Utah [Dissertation]. Graduate School of Social Work, University of Utah.

[cl21420-bib-0006] Bosquet Enlow, M. , Egeland, B. , Blood, E. A. , Wright, R. O. , & Wright, R. J. (2012). Interpersonal trauma exposure and cognitive development in children to age 8 years: A longitudinal study. Journal of Epidemiology and Community Health, 66(11), 1005–1010. 22493459 10.1136/jech-2011-200727 PMC3731065

[cl21420-bib-0007] Breslau, N. , Kessler, R. C. , Chilcoat, H. D. , Schultz, L. R. , Davis, G. C. , & Andreski, P. (1998). Trauma and posttraumatic stress disorder in the community: The 1996 Detroit Area Survey of Trauma. Archives of General Psychiatry, 55(7), 626–632. 9672053 10.1001/archpsyc.55.7.626

[cl21420-bib-0008] Canadian Council for Refugees . (2012). Concerns about changes to the refugee determination system. Canadian Council for Refugees.

[cl21420-bib-0009] Carswell, K. , Blackburn, P. , & Barker, C. (2011). The relationship between trauma, post‐migration problems and the psychological well‐being of refugees and asylum seekers. International Journal of Social Psychiatry, 57(2), 107–119. 21343209 10.1177/0020764009105699

[cl21420-bib-0010] Cleveland, J. , & Rousseau, C. (2013). Psychiatric symptoms associated with brief detention of adult asylum seekers in Canada. The Canadian Journal of Psychiatry, 58(7), 409–416. 23870723 10.1177/070674371305800706

[cl21420-bib-0011] Cleveland, J. , Rousseau, C. , & Kronick, R. (2012a). Bill C‐4: The impact of detention and temporary status on asylum seekers' mental health. Brief for submission to the House of Commons Committee on Bill C‐4, the Preventing Human Smugglers from Abusing Canada's Immigration System Act.

[cl21420-bib-0012] Cleveland, J. , Rousseau, C. , & Kronick, R. (2012b). The harmful effects of detention and family separation on asylum seekers' mental health in the context of Bill C‐31. Brief submitted to the House of Commons Standing Committee on Citizenship and Immigration concerning Bill C‐31, the Protecting Canada's Immigration System Act. 20121–20.

[cl21420-bib-0013] Coffey, G. J. , Kaplan, I. , Sampson, R. C. , & Tucci, M. M. (2010). The meaning and mental health consequences of long‐term immigration detention for people seeking asylum. Social Science & Medicine (1982), 70(12), 2070–2079. 20378223 10.1016/j.socscimed.2010.02.042

[cl21420-bib-0014] Commonwealth Immigration Ombudsman . (2024). Final report. Suicide and self‐harm in the immigration detention network. http://www.ombudsman.gov.au/__data/assets/pdf_file/0022/30298/December-2013-Suicide-and-self-harm-in-the-Immigration-Detention-Network.pdf

[cl21420-bib-0015] Council of Europe . (2010). The detention of asylum seekers and irregular migrants in Europe. Report, Committee on Migration, Refugees and Population.

[cl21420-bib-0016] Council of Europe . (2019) Alternatives to immigration detention: Fostering effective results. PRACTICAL GUIDE: Adopted at the 91th CDDH meeting.

[cl21420-bib-0017] Derrick, S. , Ingrid, S. , Annette, F. , Vijaya, M. , & Zachary, S. (1997). Anxiety, depression and PTSD in asylum‐seekers: Associations with pre‐migration trauma and post‐migration stressors. The British Journal of Psychiatry, 170, 351. 9246254 10.1192/bjp.170.4.351

[cl21420-bib-0018] DerSimonian, R. , & Laird, N. (1986). Meta‐analysis in clinical trials. Controlled Clinical Trials, 7, 177–188. 3802833 10.1016/0197-2456(86)90046-2

[cl21420-bib-0019] Essex, R. , Kalocsányiová, E. , Young, P. , & McCrone, P. (2022). Psychological distress in Australian onshore and offshore immigration detention centres from 2014–2018. Journal of Immigrant and Minority Health, 24(4), 868–874. 10.1007/s10903-022-01335-7 35113325 PMC9256570

[cl21420-bib-0020] Fazel, M. , & Stein, A. (2004). UK immigration law disregards the best interests of children. The Lancet, 363(9423), 1749–1750. 10.1016/S0140-6736(04)16337-X 15172770

[cl21420-bib-0021] Fell, P. , & Fell, B. (2010). Social work with asylum seekers and refugees: Making a difference. Policy.

[cl21420-bib-0022] Filges, T. , Lindstrøm, M. , Montgomery, E. , Kastrup, K. , & Jørgensen, A. M. K. (2014). PROTOCOL: The impact of detention on the health of asylum seekers: A protocol for a systematic revi ew. Campbell Systematic Reviews, 10(1), 1–51. 10.1002/CL2.187

[cl21420-bib-0023] Filges, T. , Montgomery, E. , & Kastrup, M. (2018). The impact of detention on the health of asylum seekers: A systematic review. Research on Social Work Practice, 28(4), 399–414. 10.1177/1049731516630384

[cl21420-bib-0024] Filges, T. , Montgomery, E. , Kastrup, M. , & Jørgensen, A.‐M. K. (2015). The impact of detention on the health of asylum seekers: A systematic review. Campbell Systematic Reviews, 11(1), 1–105. 10.4073/csr.2015.13 PMC11228430 38982995

[cl21420-bib-0025] Forrest, W. , & Steel, Z. (2023). The impact of immigration detention on the mental health of refugees and asylum seekers. Journal of Traumatic Stress, 36(3), 642–653. 10.1002/jts.22944 37338992

[cl21420-bib-0026] Gavranidou, M. , & Rosner, R. (2003). The weaker sex? Gender and post‐traumatic stress disorder. Depression and Anxiety, 17(3), 130–139. 12768647 10.1002/da.10103

[cl21420-bib-0027] Halligan, S. L. , & Rachel, Y. (2000). Risk factors for PTSD. PTSD Research Quarterly, 11(3), 1–3.

[cl21420-bib-0028] Hedrick, K. , Armstrong, G. , Coffey, G. , & Borschmann, R. (2019). Self‐harm in The Australian asylum seeker population: A national records‐based study. SSM – Population Health, 8, 100452. 31440577 10.1016/j.ssmph.2019.100452 PMC6698923

[cl21420-bib-0029] Higgins, J. P. T. (2003). Measuring inconsistency in meta‐analyses. BMJ, 327(7414), 557–560. 12958120 10.1136/bmj.327.7414.557 PMC192859

[cl21420-bib-0030] Higgins, J. P. T. , Altman, D. G. , Gotzsche, P. C. , Juni, P. , Moher, D. , Oxman, A. D. , Savovic, J. , Schulz, K. F. , Weeks, L. , & Sterne, J. A. C. (2011). The Cochrane collaboration's tool for assessing risk of bias in randomised trials. BMJ, 343, d5928. 22008217 10.1136/bmj.d5928 PMC3196245

[cl21420-bib-0031] Higgins, J. P. T. , & Sally, G. (2011). Cochrane handbook for systematic reviews of interventions. Wiley Online Library.

[cl21420-bib-0032] Higgins, J. P. T. , Savović, J. , Page, M. J. , & Sterne, J. A. C. (Eds., on behalf of the ROB2 Development Group). (2019). Revised Cochrane risk‐of‐bias tool for randomized trials (RoB 2): Detailed guidance.

[cl21420-bib-0033] Hughes, J. , & Liebaut, L. (1998). Detention of asylum seekers in Europe: Analysis and perspectives (Vol. 1). Kluwer Law International.

[cl21420-bib-0034] Human Rights and Equal Opportunity Commission . (1998). Those who've come across the seas: Detention of unauthorised arrivals. Retrieved June 18, 2015, from: http://www.hreoc.gov.au/pdf/human_rights/asylum_seekers/h5_2_2.pdf

[cl21420-bib-0035] Human Rights Watch . (2001). No safe refuge: The impact of the September 11 attacks on refugees, asylum seekers and migrants in the Afghanistan region and worldwide. Retrieved January 19, 2012, from: http://www.hrw.org/backgrounder/refugees/refugee-bck1017.pdf

[cl21420-bib-0036] Ichikawa, M. , Nakahara, S. , & Wakai, S. (2006). Effect of post‐migration detention on mental health among Afghan asylum seekers in Japan. Australian and New Zealand Journal of Psychiatry, 40, 341–346. 16620316 10.1080/j.1440-1614.2006.01800.x

[cl21420-bib-0037] Janet, P. , & Harriet, S . (2013). Boat arrivals in Australia since 1976. Parliamentary Library.

[cl21420-bib-0038] Janoff‐Bulman, R. (1992). Shattered assumptions: Toward a new psychology of trauma. Free Press.

[cl21420-bib-0040] Jesuit Refugee Service Europe . (2013). JRS Europe Policy position on alternatives to detention. Retrieved September 2012.

[cl21420-bib-0041] Jo, P. , & Rachel, J. (2002). Mental health primary care in prison: A guide to mental ill health in adults and adolescents in prison and young offender institutions. Royal Society of Medicine Press.

[cl21420-bib-0042] Johnston, V. , Allotey, P. , Mulholland, K. , & Markovic, M. (2009). Measuring the health impact of human rights violations related to Australian asylum policies and practices: A mixed methods study. BMC International Health and Human Rights, 9, 1–12. 19192307 10.1186/1472-698X-9-1 PMC2649030

[cl21420-bib-0043] Karlsen, E. (2016–17). Australia's offshore processing of asylum seekers in Nauru and PNG: A quick guide to statistics and resources. Parliament of Australia. RESEARCH PAPER SERIES, 20161–16.

[cl21420-bib-0044] Keith, C. W. , Brunell, A. B. , & Foster, J. D. (2004). Sitting here in limbo: Ego shock and posttraumatic growth. Psychological Inquiry, 15(1), 22–26.

[cl21420-bib-0045] Keller, A. S. , Rosenfeld, B. , Trinh‐Shevrin, C. , Meserve, C. , Sachs, E. , Leviss, J. A. , Singer, E. , Smith, H. , Wilkinson, J. , Kim, G. , Allden, K. , & Ford, D. (2003). Mental health of detained asylum seekers. The Lancet, 362, 1721–1723. 10.1016/S0140-6736(03)14846-5 14643122

[cl21420-bib-0046] Kessler, R. C. (1995). Posttraumatic stress disorder in the National Comorbidity Survey. Archives of General Psychiatry, 52(12), 1048–1060. 7492257 10.1001/archpsyc.1995.03950240066012

[cl21420-bib-0047] Kirmayer, L. J. (1996). Confusion of the senses: Implications of ethnocultural variations in somatoform and dissociative disorders for PTSD. In A. J. Marsella , M. J. Friedman , E. T. Gerrity , & R. M. Scurfield (Eds.), Ethnocultural aspects of posttraumatic stress disorder: Issues, research, and clinical applications (pp. 131–163). American Psychological Association.

[cl21420-bib-0048] Koopowitz, L. F. , & Abhary, S. (2004). Psychiatric aspects of detention: Illustrative case studies. The Australian and New Zealand Journal of Psychiatry, 38(7), 495–500. 15255821 10.1080/j.1440-1614.2004.01402.x

[cl21420-bib-0049] Lacroix, M. (2006). Social work with asylum seekers in Canada: The case for social justice. International Social Work, 49(1), 19–28.

[cl21420-bib-0050] Lewis, H. J. (1997). *Trauma and recovery: The aftermath of violence—From domestic abuse to political terror*. Hachette Book Group.

[cl21420-bib-0039] Lifton, R. J. (1993). From Hiroshima to the Nazi doctors: The evolution of psychoformative approaches to understanding traumatic stress syndromes. In J. P. Wilson & B. Raphael (Eds.), International handbook of traumatic stress syndromes (pp. 11–23). Springer.

[cl21420-bib-0051] Lipsey, M. W. , & Wilson, D. B. (2001). Practical meta‐analysis. Sage Publications Inc.

[cl21420-bib-0052] Loff, B. (2002). Detention of asylum seekers in Australia. The Lancet, 359(9308), 792–793. 10.1016/S0140-6736(02)07887-X 11888610

[cl21420-bib-0053] Lustig, S. , Kia‐Keating, M. , Grant‐Knight, W. , Geltman, P. , Ellis, H. , Birman, D. , & Saxe, G. N. (2003). Review of child and adolescent refugee mental health: White paper from the National Child Traumatic Stress Network. US Department of Health and Human Services: Substance Abuse and Mental Health Services Administration.

[cl21420-bib-0054] Mace, A. O. , Mulheron, S. , Jones, C. , & Cherian, S. (2014). Educational, developmental and psychological outcomes of resettled refugee children in Western Australia: A review of school of special educational needs: Medical and mental health input. Journal of Paediatrics and Child Health, 50(12), 985–992. 10.1111/jpc.12674 24976219

[cl21420-bib-0055] Mares, S. (2021). Mental health consequences of detaining children and families who seek asylum: A scoping review. European Child & Adolescent Psychiatry, 30, 1615–1639. 10.1007/s00787-020-01629-x 32926223

[cl21420-bib-0056] McColl, H. , McKenzie, K. , & Bhui, K. (2008). Mental healthcare of asylum‐seekers and refugees. Advances in Psychiatric Treatment, 14(6), 452–459.

[cl21420-bib-0057] Medical Foundation for the Care of Victims of Torture . (1994). A betrayal of hope and trust: Detention in the UK of survivors of torture. Medical Foundation London.

[cl21420-bib-0058] Michael, W. , & Liza, S. (2005). Detention of asylum seekers in the UK and USA: Deciphering noisy and quiet constructions. Punishment & Society, 7(4), 397–417.

[cl21420-bib-0059] Mina, F. , & Derrick, S. (2006). Detention of refugees. BMJ, 332(7536), 251–252. 16455700 10.1136/bmj.332.7536.251 PMC1360383

[cl21420-bib-0060] Mollica, R. F. , Caspi‐Yavin, Y. , Bollini, P. , Truong, T. , Tor, S. , & Lavelle, J. (1992). The Harvard Trauma Questionnaire. Validating a cross‐culturalinstrument for measuring torture, trauma, and post‐traumaticstress disorder in Indochinese refugees. The Journal of Nervous and Mental Disease, 180, 111–116. 1737972

[cl21420-bib-0061] Momartin, S. , Steel, Z. , Coello, M. , Aroche, J. , Silove, D. M. , & Brooks, R. (2006). A comparison of the mental health of refugees with temporary versus permanent protection visas. Medical Journal of Australia, 185, 357–361. 17014402 10.5694/j.1326-5377.2006.tb00610.x

[cl21420-bib-0062] National Legislative Bodies/National Authorities . (2013). Regional resettlement arrangement between Australia and Papua New Guinea [Australia]. Retrieved November 13, 2013, from: http://www.refworld.org/docid/51f61a504.html

[cl21420-bib-0063] Nyers, P. (2003). Abject cosmopolitanism: The politics of protection in the anti‐deportation movement. Third World Quarterly, 24(6), 1069–1093.

[cl21420-bib-0064] Patel, V. (1995). Spiritual distress: An indigenous model of nonpsychotic mental illness in primary care in Harare, Zimbabwe. Acta Psychiatrica Scandinavica, 92(2), 103–107. 7572254 10.1111/j.1600-0447.1995.tb09551.x

[cl21420-bib-0065] Phillips, J. (2012). The ‘Pacific Solution’ revisited: A statistical guide to the asylum seeker caseloads on Nauru and Manus Island Janet Phillips Social Policy Section. Parliament of Australia BACKGROUND NOTE. 20121–20.

[cl21420-bib-0066] Physicians for Human Rights and the Bellevue/NYU Program for Survivors of Torture . (2003). From persecution to prison: The health consequences of detention for asylum seekers. http://www.physiciansforhumanrights.org/library/documents/reports/report-perstoprison-2003.pdf

[cl21420-bib-0067] Pourgourides, C. (1997). A second exile: The mental health implications of detention of asylum seekers in the UK. Psychiatric Bulletin, 21(11), 673–674.

[cl21420-bib-0068] Pourgourides, C. , Sashidharans, P. , & Bracken, P. J. (1996). A second exile: The mental health implications of detention of asylum seekers in the UK. North Birmingham Mental Health Trust.

[cl21420-bib-0069] Pratchett, L. C. , Pelcovitz, M. R. , & Yehuda, R. (2010). Trauma and violence: Are women the weaker sex? Psychiatric Clinics of North America, 33(2), 465–474. 20385347 10.1016/j.psc.2010.01.010

[cl21420-bib-0070] Robjant, K. Psychological distress of asylum seekers in immigration detention [PhD Thesis]. University of Surrey (United Kingdom) 2007, 1–216.

[cl21420-bib-0071] Robjant, K. , Hassan, R. , & Katona, C. (2009). Mental health implications of detaining asylum seekers: Systematic review. British Journal of Psychiatry, 194(4), 306–312. 10.1192/bjp.bp.108.053223 19336779

[cl21420-bib-0072] Robjant, K. , Robbins, I. , & Senior, V. (2009). Psychological distress amongst immigration detainees: A cross‐sectional questionnaire study. British Journal of Clinical Psychology, 48, 275–286. 19200410 10.1348/014466508X397007

[cl21420-bib-0073] Rowcliffe, C. , Stellenberg, R. , & Cherian, S. (2016). The impact of detention on children and adolescents. Journal of Paediatrics and Child Health, 52(9), 912–913. 10.1111/jpc.13282 27650157

[cl21420-bib-0074] Salinsky, M. (1997). Detaining asylum seekers. BMJ, 314(7079), 456. 9056786 10.1136/bmj.314.7079.456 PMC2125980

[cl21420-bib-0075] Sánchez‐Meca, J. , Marín‐Martínez, F. , & Chacón‐Moscoso, S. (2003). Effect‐size indices for dichotomized outcomes in meta‐analysis. Psychological Methods, 8(4), 448–467. 14664682 10.1037/1082-989X.8.4.448

[cl21420-bib-0076] Schuster, L. (2004). The exclusion of asylum seekers in Europe. centre on migration. Policy and Society Working Paper, 1, 1–22.

[cl21420-bib-0077] Silove, D. , Steel, Z. , McGorry, P. , & Mohan, P. (1998). Trauma exposure, postmigration stressors, and symptoms of anxiety, depression and post‐traumatic stress in Tamil asylum‐seekers: Comparison with refugees and immigrants. Acta Psychiatrica Scandinavica, 97(3), 175–181. 9543304 10.1111/j.1600-0447.1998.tb09984.x

[cl21420-bib-0078] Silove, D. , Steel, Z. , & Mollica, R. F. (2001). Detention of asylum seekers: Assault on health, human rights, and social development. The Lancet, 357(9266), 1436–1437. 10.1016/s0140-6736(00)04575-x 11356469

[cl21420-bib-0079] Silove, D. , Steel, Z. , & Watters, C. (2000). Policies of deterrence and the mental health of asylum seekers. Journal of the American Medical Association, 284(5), 604–611. 10918707 10.1001/jama.284.5.604

[cl21420-bib-0080] Sinnerbrink, I. , Silove, D. , Field, A. , Steel, Z. , & Manicavasagar, V. (1997). Compounding of premigration trauma and postmigration stress in asylum seekers. The Journal of Psychology, 131(5), 463–470. 9284551 10.1080/00223989709603533

[cl21420-bib-0081] Steel, Z. , Momartin, S. , Silove, D. , Coello, M. , Aroche, J. , & Tay, K. W. (2011). Two year psychosocial and mental health outcomes for refugees subjected to restrictive or supportive immigration policies. Social Science & Medicine (1982), 72, 1149–1156. 21427011 10.1016/j.socscimed.2011.02.007

[cl21420-bib-0082] Steel, Z. , Silove, D. , Brooks, R. , Momartin, S. , Alzuhairi, B. , & Susljik, I. (2006). Impact of immigration detention and temporary protection on the mental health of refugees. British Journal of Psychiatry, 188, 58–64. 10.1192/bjp.bp.104.007864 16388071

[cl21420-bib-0083] Sterne, J. A. , Hernán, M. A. , Reeves, B. C. , Savović, J. , Berkman, N. D. , Viswanathan, M. , & Higgins, J. P. (2016). ROBINS‐I: A tool for assessing risk of bias in non‐randomized studies of interventions. BMJ, 355, 1–7. 10.1136/bmj.i4919 PMC5062054 27733354

[cl21420-bib-0084] Sterne, J. A. C. , Higgins, J. P. T. , Elbers, R. G. , & Reeves, B. C. (2016). The development group for ROBINS‐I. Risk of bias in non‐randomized studies of interventions (ROBINS‐I): Detailed guidance, WP Updated (pp. 1–53).

[cl21420-bib-0085] Summerfield, D. , Gorst‐Unsworth, C. , Bracken, P. , Tonge, V. , Forrest, D. , & Hinshelwood, G. (1991). Detention in the UK of tortured refugees. The Lancet, 338(8758), 58. 10.1016/0140-6736(91)90051-p 1676113

[cl21420-bib-0086] Tania, S. , & Marianne, E. (2013). The impact of immigration detention on the mental health of torture survivors is poorly documented—A systematic review. Danish Medical Journal, 60(11), A4728. 24192244

[cl21420-bib-0087] The Information Centre about Asylum and Refugees (ICAR) . (2007). Detention of asylum seekers in the UK (2007). Thematic Briefing prepared for the Independent Asylum Commission Information Centre about Asylum and Refugees (ICAR). Retrieved from: http://www.icar.org.uk/Detention_of_asylum_seekers_in_the_UK_June_2007.pdf

[cl21420-bib-0088] Thompson, M. (2011). The Psychological Impact of Torture and Other Types of Systemic Abuse – Examining permanent residents and asylum seekers in the community and in detention in Australia [Ph.D. Thesis. Department of Psychology, The University of Melbourne].

[cl21420-bib-0089] Thompson, M. , McGorry, P. , Silove, D. , & Steel, Z. (1998). Maribyrnong detention centre tamil survey. In D. Silove , & Z. Steel (Eds.), The mental health and well‐being of on‐shore asylum seekers in Australia (1 ed., pp. 27–31). University of New South Wales, Psychiatry Research & Teaching Unit.

[cl21420-bib-0090] UN High Commissioner for Refugees (UNHCR) . (1999). Revised guidelines on applicable criteria and standards relating to the detention of asylum‐seekers. United Nations High Commissioner for Refugees.

[cl21420-bib-0091] United Nations . (2000). High commissioner for refugees. The state of the world's refugees: A humanitarian agenda, United Nations High Commission for Refugees.

[cl21420-bib-0092] United Nations High Commissioner for Refugees . (1999a). Detention of asylum‐seekers and refugees: The framework, the problem and recommended practice. Conference room paper, EC/49/SC/CRP.13.

[cl21420-bib-0093] United Nations High Commissioner for Refugees . (1999b). UNHCR's Guidelines on applicable criteria and standards relating to the detention of asylum‐seekers. United Nations High Commissioner for Refugees.

[cl21420-bib-0094] United Nations High Commissioner for Refugees . (2010). Convention and protocol relating to the status of refugees. United Nations High Commission for Refugees.

[cl21420-bib-0095] United Nations High Commissioner for Refugees . (2013). Australia–Papua New Guinea asylum agreement presents protection challenges. United Nations High Commissioner for Refugees.

[cl21420-bib-0096] Vikram, P. , Essie, S. , & Fungisai, G. (1995). Kufungisisa (thinking too much): A Shona idiom for non‐psychotic mental illness. The Central African Journal of Medicine, 41(7), 209–215. 7553793

[cl21420-bib-0097] von Werthern, M. , Robjant, K. , Chui, Z. , Schon, R. , Ottisova, L. , Mason, C. , & Katona, C. (2018). The impact of immigration detention on mental health: A systematic review. BMC Psychiatry, 18(382), 382. 10.1186/s12888-018-1945-y 30522460 PMC6282296

[cl21420-bib-0098] Zur, J. (1996). From PTSD to voices in context from an “experience‐far” to an “experience‐near” understanding of responses to war and atrocity across cultures. International Journal of Social Psychiatry, 42(4), 305–317. 9023611 10.1177/002076409604200405

[cl21420-bib-0099] Zwi, K. , Mares, S. , Nathanson, D. , Tay, A. K. , & Silove, D. (2018). The impact of detention on the social‐emotional wellbeing of children seeking asylum: A comparison with community‐based children. European Child & Adolescent Psychiatry, 27(4), 411–422. 10.1007/s00787-017-1082-z 29177563

