# Supplementary material for: The impact of detention on the health of asylum seekers: An updated systematic review: A systematic review
Source: Campbell Syst Rev. 2024 Jul 8;20(3):e1420. doi: 10.1002/cl2.1420 (PMC11228430; doi:10.1002/cl2.1420)
Supplement: Supplementary file 5 — Supporting information. [file CL2-20-e1420-s001.docx]

**Supplementary materials**

[For display in the published PDF only] Supplementary materials are available with the online version of this article: 10.1002/14651858.CA000411.

[For display on the Cochrane Library only] Supplementary materials are published alongside the article and contain additional data and information that support or enhance the article. Supplementary materials may not be subject to the same editorial scrutiny as the content of the article and Cochrane has not copyedited, typeset or proofread these materials. The material in these sections has been supplied by the author(s) for publication under a Licence for Publication and the author(s) are solely responsible for the material. Cochrane accordingly gives no representations or warranties of any kind in relation to, and accepts no liability for any reliance on or use of, such material.

Supplementary material 1

CA000411-SUP-01-searchStrategy.html

Search strategies

Supplementary material 2

CA000411-SUP-02-characteristicsOfIncludedStudies.html

Characteristics of included studies

Supplementary material 3

CA000411-SUP-03-characteristicsOfExcludedStudies.html

Characteristics of excluded studies

Supplementary material 4

CA000411-SUP-04-characteristicsOfAwaitingStudies.html

Characteristics of studies awaiting classification

Supplementary material 5

CA000411-SUP-05-dataPackage.zip

Data package
